# A review of gorgonian coral species (Cnidaria, Octocorallia, Alcyonacea) held in the Santa Barbara Museum of Natural History research collection: focus on species from Scleraxonia, Holaxonia, and Calcaxonia – Part I: Introduction, species of Scleraxonia and Holaxonia (Family Acanthogorgiidae)

**DOI:** 10.3897/zookeys.860.19961

**Published:** 2019-07-04

**Authors:** Elizabeth Anne Horvath

**Affiliations:** 1 Westmont College, 955 La Paz Road, Santa Barbara, California 93108 USA Westmont College Santa Barbara United States of America; 2 Invertebrate Laboratory, Santa Barbara Museum of Natural History, 2559 Puesta del Sol Road, Santa Barbara, California 93105, USA Santa Barbara Museum of Natural History Santa Barbara United States of America

**Keywords:** Allan Hancock Foundation (AHF) – ‘Velero’ Expeditions, museum collection, new species, “red whips”, soft corals, *
Swiftia
*, “thread-like” forms

## Abstract

Gorgonian specimens collected from the California Bight (northeastern Pacific Ocean) and adjacent areas held in the collection of the Santa Barbara Museum of Natural History (SBMNH) were reviewed and evaluated for species identification; much of this material is of historic significance as a large percentage of the specimens were collected by the Allan Hancock Foundation (AHF) ‘Velero’ Expeditions of 1931–1941 and 1948–1985. Examination and reorganization of this collection began early in 2002; initially, it was estimated that at most, twelve to fifteen species of gorgonian could be found within the Bight. Following collection evaluation, it was determined that at a minimum, approximately twenty three genera, encompassing some forty-plus species, of gorgonian coral have been found living within the California Bight region, often extending some distance into adjacent geographical areas both north and south. All species from the California Bight in the collection are discussed to some degree (in three separate parts, this being Part I), with digital images of both colony form and sclerite composition provided for most. Collection specimens from the suborders and families covered in Part I are not extensive, but several genera are featured that have not been previously reported for the California Bight region. Additionally, a potential new species (genus *Sibogagorgia* Stiasny, 1937) from the Paragorgiidae is described in Part I. Overall, the collection displays an emphasis on species belonging to the Holaxonia, particularly the plexaurids. A brief discussion of a California Bight grouping, referred to as the “red whips,” is presented in Part II; this grouping encompasses several species with very similar colony appearance across a number of genera. A new species (a whip or thread-like form) in the genus *Eugorgia* Verrill, 1868, belonging to the Gorgoniidae, is described in Part II. The genus *Swiftia* Duchassaing & Michelotti, 1864 is one of the most challenging taxon groups represented; those species in the genus *Swiftia* collected within the California Bight are discussed fully, based on SBMNH (and other) specimens in Part III. Scanning electron microscopy images for species of *Swiftia* from the California coast have rarely, if ever, been published and are included, with a discussion of the geographic range of the genus in the eastern Pacific, from the southern boundary of the California Bight to the Bering Sea, Alaska. Finally, specimens of the genus *Thesea* Duchassaing & Michelotti, 1860, displaying a whip or thread-like body form, are discussed at a preliminary level in Part III; they also presented challenges to a clear understanding of their taxonomy. While Part I focuses on species of Scleraxonia and those of the Holaxonia in the Acanthogorgiidae family held in the SBMNH collection, all three parts taken together represent the first comprehensive work that reviews the research collection of SBMNH, which focuses on species of gorgonian coral known to inhabit the California Bight.

*The trawl seemed to have gone over a regular field of a delicate, simple Gorgonid . . . . the stems, which were from eighteen inches to two feet in length, were coiled in great hanks around the beam-trawl and engaged in masses in the net; and as they showed a most vivid phosphorescence of a pale lilac colour, their immense number suggested a wonderful slate of things beneath – animated cornfields waving gently in a slow tidal current and glowing with a soft diffused light, scintillating and sparkling on the slightest touch, and now and again breaking into long avenues of vivid light indicating the paths of fishes or other wandering denizens of their enchanted region.* Wyville Thomson (during the voyage of the ‘Challenger’)

## Introduction

With an extensive collection of California gorgonians housed at Scripps Institute of Oceanography (Univ. of California, San Diego) to the south and a large gorgonian collection housed to the north (California Academy of Sciences, San Francisco), the Santa Barbara Museum of Natural History (SBMNH) research collection would seem to be a small and redundant collection. However, SBMNH is the repository for the bulk of the Allan Hancock Foundation (AHF) cnidarian collection, amassed from the ‘Velero’ Expeditions (1931–1941, 1948–1985). Thus, the SBMNH research collection includes many gorgonian specimens of historical interest. It is a significant resource for those studying this cnidarian group as it may best illustrate the diversity of gorgonian species that can be found in California waters. This is the first part of a comprehensive work that reviews the research collection of SBMNH, which focuses on species of gorgonian coral known to inhabit the California Bight.

Brief taxonomic reviews of previously described species (with representation in the collection), and additional information on species recorded from the Bight, regarding distribution, biology/ecology and noteworthy observations are presented in all three parts. Also included are more extensive descriptions for genera and species not previously reported for the California Bight, as are fuller descriptions for several potentially new species. It is apparent from the SBMNH research collection that gorgonian coral diversity within the California Bight is far greater than previously thought, pointing to a geographic location that needs further extensive exploration throughout, both geographically and with depth.

For the purposes of understanding what is meant by a “gorgonian,” as discussed in all three parts of this work, the term “gorgonian” is used in reference to members of the Subclass Octocorallia, Order Alcyonacea, specifically the scleraxonians, holaxonians, and calcaxonians. Defining features (based on Bayer 1981c, Fabricius and Alderslade 2001, Brusca et al. 2016) include: 1) modular colonies, either encrusting or erect, often massive in size, usually colorful, 2) with colonies generally branched and tree-like, often forming flat, fan-like shapes, 3) stems and branches with a stiff, supporting, organic (horny), centrally internal skeletal axis (whether or not enclosed by a specialized secretory epithelium), comprised of calcite and scleroproteinous gorgonin, producing a relatively solid central support (scleraxonians with axis of fused sclerites, holaxonians with axis having no free sclerites but with hollow, cross-chambered central core and calcaxonians without hollow, cross-chambered central core but with large amounts of nonscleritic CaCo_3_ forming internodes), with 4) polyps each supported by a portion of the central axis and having 5) all portions of central axis covered with a fleshy and flexible, somewhat thin coenenchyme, filled with embedded and surface sclerites. The gorgonian octocorals of the California Bight discussed herein (and subsequent parts) are those commonly referred to as sea fans and sea whips.

The subclass is composed of 50 families (Williams and Cairns 2014), with the gorgonians themselves encompassing 16–19 of those families, comprising more than 3,400 species (Daly et al. 2007, McFadden and Ofwegen 2013, Williams and Cairns 2014). Gorgonian corals can be found in all oceans (McFadden and Ofwegen 2013) from low inter-tidal to extreme depth (Williams 1990, 2013), varying widely in size, color and branch pattern. While gorgonian corals are known in a general sense by many who snorkel or scuba-dive, these organisms often do not readily identify to species based on the field identifiers of size, color, or branch morphology. Taxonomic revisions of numerous genera (such as Breedy and Guzmán 2007, 2011, 2016; Breedy et al. 2009) are necessary before many species can be recognized as valid and correctly identified, due to lack of clarity in many of the older original descriptions.

While seemingly fragile and delicate in overall appearance, gorgonians are remarkably “plastic” (Bayer 1958, 1961, Grigg 1972, Muzik 1979, Fabricius and Alderslade 2001, Horvath 2011). They exhibit an array of body forms and a rather hardy lifestyle, either living at great depth (a hostile environment of intense cold and pressure with no light) (Freiwald and Roberts 2005, Watling et al. 2011) or in shallow, sunlit climes, in areas with slow, constant current, or in areas of marked current flow, literally assuming a stance that allows them maximum exposure to that current (Wainwright and Dillon 1969, Grigg 1970, 1972, Leversee 1976, DeVogelaere et al. 2005). Some deep-water species achieve great size (Heifetz 2002, DeVogelaere et al. 2005) and longevity (Grigg 1974, 1975, Griffen and Druffel 1989, Druffel et al. 1990, 1995, Risk et al. 2002, Andrews et al. 2005, Sherwood et al. 2005), all produce an interesting array of organic compounds used for defense, some of which may prove to be pharmaceutical in nature (Fenical et al. 1981, Rodriquez et al. 1994, Deghrigue et al. 2013, Almeida et al. 2014), and some exhibit an amazingly wide range of geographic distribution. Many seem to function as a sheltering, protective, three-dimensional “tree,” harboring many other forms of organism, in or under their branches (Buhl-Mortensen and Mortensen 2004a, 2004b, Auster 2005, Devogelaere et al. 2005, Etnoyer and Morgan 2005, Brancato et al. 2007, Buhl-Mortensen et al. 2010, Rossi et al. 2017). Those species that prefer warm water are often the conspicuous fan-like forms noted in context with stony, reef-building corals, adding a brilliant array of color to the reef. Others, preferring temperate water, may be only generally known in some areas (particularly true of those species with representation in the SBMNH collection seen in the California Bight, which are usually identified by eye, and seemingly not very diverse with regards to number of species), and still others, preferring extreme cold and often seen at great depth, are species that are surprising researchers with their level of diversity, abundance and color. The focus here is on temperate water species found in the research collection at SBMNH from the California Bight (and immediately adjacent areas). Initially, the collection appeared to be composed of a fairly consistent group of gorgonian corals, represented by a dozen or so species that many working within the Bight seemed to know. A primary goal was to assess the SBMNH research collection, and while conducting the reorganization of the collection, to confirm or refute the assertion of such a small number of species in a region that is known for its high degree of biodiversity (McGinnis 2006).

The primary method used for identification of a gorgonian coral species is examination of the skeletal structures embedded in the gorgonian’s soft tissue; identification in this group has always required microscopic examination of the skeletal elements, the sclerites (Valenciennes 1855, Grasshoff and Bargibant 2001). The importance of the sclerite to species identification was first recognized by Valenciennes in 1855, although he often neglected to put his observations into practice. Kölliker (1865) put this means of identification on stronger footing and gorgonian taxonomists following in his stead (Verrill 1868, Kükenthal 1924) adopted, expanded and improved the system further; this system was finally emended by Hickson in 1930 (Hickson 1930). Further work by Bayer, dating from the late 1940s firmly established this practice and continues to be the best morphological method for determining species identification. While branch pattern, size of colony, and its color can be useful features in the identification of a gorgonian octocoral, particularly when working in the field or when doing a first examination of a collected specimen, these characters only provide limited identification. With these characters only, determination to a genus might be possible, but ultimately, the single most important character in octocoral identification is the microscopic calcareous sclerite. Sclerites, formerly known as spicules (Bayer et al. 1983), are found in nearly all species of octocoral (Fabricius and Alderslade 2001). Found in large number in every colony, “ten’s of thousands (would) not (be) unusual in a modest-sized colony. Even a small fragment may contain thousands of sclerites” (Cairns, in Etnoyer et al. 2006). These structures, composed of calcitic calcium carbonate, range in size from twenty µm to five mm and are found in both polyps and the coenenchymal tissue between the polyps (Cairns, in Etnoyer et al. 2006). These skeletal structures provide a small measure of support and structure to polyps and their tissue (Lewis and von Wallis 1991). As can be seen in the illustrated trilingual glossary, published in 1983, edited by Bayer, Grasshoff, and Verseveldt, sclerites themselves show an amazing range of diversity in both size and shape. At first, the variety of sclerite shapes can seem overwhelming, and the names given for sclerite forms rather fanciful (torch vs. leaf-scale vs. caterpillar vs. opera glass) (Bayer et al. 1983). This glossary, a first attempt at classifying all the possible forms of sclerite seen in octocorals, ultimately defined, synonymized, and illustrated 57 different sclerite forms. Correct identification of specimens in the SBMNH collection, using sclerite characters, was a key component of the reorganization undertaken.

Other tools that are currently being explored and utilized, with varying degrees of success, for potential identification of organisms are genetic methods and molecular analysis (Etnoyer et al. 2006). Various octocorals, including alcyonacean gorgonians, have been examined by this means (Berntson et al. 2001, France and Hoover 2001, Sánchez et al. 2003a, 2003b, McFadden et al. 2006, Sánchez et al. 2007, Herrera et al. 2010, McFadden et al. 2011, Quattrini et al. 2014, Figueroa and Baco 2014a). These efforts, while still in their infancy, may add valuable insight into the relationships between, and evolutionary development of, gorgonian octocorals, with their taxonomic standing, but will not supplant the need for direct examination of colonies and their sclerites.

As study and reorganization of the SBMNH collection began, it was suggested that identification of gorgonians from a study of their defensive nematocysts might be possible; some work had been conducted that illustrated possible mechanisms for nematocyst function (Mariscal and Bigger 1976, Sebens and Miles 1988), but no published taxonomic work using nematocysts as characters for identification could be found. Successful identification efforts were best achieved through microscopic examination of the sclerites. Sclerites, however, are affected by current flow (both its direction and speed), and other environmental conditions, evidenced by their placement, numbers, thickness, shape variation and degree of ornamentation. This was confirmed through a number of sources (e.g., Gori et al. 2012).

A further area of study that needs to be more extensively undertaken is that regarding gorgonian coral ecology; studies that necessarily focus on the ways in which these organisms deal with the physical features of their environment, as well as the biological issues of finding food, defending themselves from predators and successfully mating. Fabricius and Alderslade (2001) provide a short but comprehensive overview of the physical and biological challenges that all octocorals must address. What we currently know about gorgonian ecology will certainly be affected in the future by the destruction of habitats (Brancato et al. 2007), changes in temperature, water pH (acidification), as discussed in a number of published articles (Orr et al. 2005, Wood et al. 2008, Yilmaz et al. 2008; Ziveri 2012, Bramanti et al. 2013), and nutrient levels resulting from global climate change, the alteration in geographic distribution of other organisms stemming from that same climatic change (and how the shifts in other animal and plant populations may affect the substrate-restricted gorgonian corals) and the on-going interest that biochemists and pharmacologists have in the gorgonians (and other invertebrate organisms). Of particular interest with regards to gorgonian ecology are the many obligate symbiont associations that gorgonians have, particularly with other species of cnidarians, cirripede barnacles, amphipods, copepods, and ophiuroids (Patton 1972, Laubitz and Lewbel 1974, Lewbel 1976, Humes and Lewbel 1977, Buhl-Mortensen and Mortensen 2004a, b), to name but a few, as well as what appear to be more symbiotic commensal relationships that exist between gorgonians and various species of fish, who may seek shelter in or under the colonies (Etnoyer and Morgan 2005, Brancato et al. 2007). There are also issues associated with predator-prey interactions for gorgonians (Gomez, 1973, Grasshoff and Bargibant 2001) as well as relationships that appear to be more parasitic in nature (McLean and Yoshioka 2007) that provide opportunities for further investigation.

Available literature on gorgonian species of the California Bight was, on one hand, somewhat abundant, but often not very accessible, being spread over so many different publications. The earliest works on California gorgonians date to those published by Verrill (1864, 1865, 1868, 1869) and Nutting (early 1900s). These older works are primarily species descriptions (many of which were designated as new species by Verrill or Nutting). Additionally, work by Kükenthal (1920s), Wright and Studer (late 1880s), Aurvillius (1930s), etc., are still available, although many of these works are in a language other than English, requiring careful translation in order to clearly determine the subtle nuances that distinguish one species from another. Unfortunately, in many of these instances, the authors were working with very small samples of very few specimens and had no context for the larger colony’s location in situ. Bayer described a large number of new species, dating from the 1950s to the present (Cairns 2009), but predominantly from geographic areas other than the California Bight. Generally, very little further taxonomic work had been published on many species from California (Hardee and Wicksten 1996); some work was carried out on the ecology and physiology of a few of the California Bight species by Grigg (1970, 1972), Lissner and Dorsey (1986), and Satterlie and Case (1978, 1979), to name the few. More recently, Williams (2013) proposed a new genus (*Chromoplexaura*) designation for a “red whip” species often found in California waters. While there have been recent reviews done by Cairns and Bayer (2005, 2009) and Cairns (2007) on the Primnoidae, Sánchez (2005) on *Paragorgia/Sibogagorgia*, and Breedy and Guzmán on *Pacifigorgia* (2002), *Leptogorgia* (2007), *Eugorgia* (2009), *Heterogorgia* (2011), and *Muricea* (2015, 2016), several of which include species from the California Bight, there was no single, comprehensive work on all gorgonian species found within the California Bight (and areas extending slightly either north or south of it), a region that is defined by Cabo Colnett, Baja, Mexico to the south and Point Arguello, California, to the north. This review of the SBMNH research collection is an attempt to fill that void. New species are likely to be described from this region in the future, and certainly, the number of possible new species that are being documented at greater depth north of the Bight, in Monterey Bay for instance, could well have implications for what potentially may still be discovered within the Bight.

The systematics of eastern Pacific coast octocorals, particularly with regards to the alcyonacean gorgonian corals from Baja, Mexico to the California, Oregon and Washington coasts, seemed sporadic and at times, unreliable. Part of the confusion generated was likely due to the cosmopolitan distribution of a number of species which, when first collected off California, were described as new species (but had actually already been described based on collection events undertaken elsewhere). As stated previously, taxonomists were often working with a very small (and not always stellar) sample from a larger colony, or did not take into account (perhaps because it was not known at the time) the environmental plasticity of these organisms; thus they would describe a species as always branching in one plane, when current flow, we now know (Wainwright and Dillon 1969, Grigg 1970, 1972, Leversee 1976) is often the reason for a colony being in more than one plane. Conversely, some specimens which appeared superficially to be the same species, had all been allocated one name when, in reality, those included in the group were actually several separate species.

A second problem related to the lack of types for some of the taxa described from the California coast. There are excellent reviews by Bayer (1956a, 1981a, 1981c) which have helped to resolve some of the generic confusion surrounding many species names that potentially represented synonyms, but there are those that still need to be resolved (Dunn, 1982). Finally, based on species being noted and/or collected off northern California (survey work being done by NOAA or the research institute associated with the Monterey Bay Aquarium, MBARI), as well as several species that independent researchers have sent to SBMNH (staff from both Los Angeles, LACSD, and Orange County OCSD, Sanitation Districts, Milton Love at University of California, Santa Barbara, etc.), some have not been described at all and it has been revealed that there are previously described species of gorgonian living in the California Bight region that have not been collected and recorded in this locality simply because they have not been looked for. Variously, these are inconspicuous colonies mistaken for some other organism, which have not been examined at the level of the sclerites, or live at depths that have only recently become accessible.

## Materials and methods

Nearly all of the specimens examined (housed currently as part of the Santa Barbara Museum of Natural History’s permanent research collection, Invertebrate Laboratory), were collected over a period of years dating from the 1930s to the present in either dry or wet condition. The collection of gorgonians housed at SBMNH, while of moderate size, is a fairly diverse one. The collection displayed diversity due to the activities of many independent collectors over the years who chose to donate their material to the museum, but the collection achieved substantial value when, in 1998, the SBMNH became the official recipient of the diverse 10,000-lot cnidarian collection, a portion of the Allan Hancock Foundation (AHF) collection built upon the historic ‘Velero’ expeditions of 1931–1941 and 1948–1985. Thus, the SBMNH cnidarian collection has become a collection of great historical significance, particularly with regards to species that live, or have lived, along northeastern Pacific shores. Of particular note were the extensive lots of material (both wet and dry) encompassing alcyonacean gorgonians, with hydroids and sea pens; these physically arrived at the museum in 2002. The SBMNH now houses approximately 515 lots (many composed of multiple specimens within a single lot) of gorgonian coral. The number of lots indicated does not include numerous lots housed elsewhere in the collection; gorgonian coral fragments are housed in the museum’s mollusk collection (the mollusks in question were found on, and collected with, a species of gorgonian, for instance), or in other sections of the museum’s cnidarian collection (such as zoanthid anemones collected on gorgonian corals). Also scattered throughout other portions of the museum’s invertebrate collection are bryozoans, barnacles, or brittle stars that were collected from within or on gorgonian coral colonies and were preserved with the gorgonian they were living with. To aid in the identification of the museum’s specimens, critical to this reorganization, examinations of specimens of known species from or collected in the Bight were also performed on material found in the collections of the National Museum of Natural History, Smithsonian (**USNM** = NMNH), the California Academy of Sciences, San Francisco (**CAS**), the Los Angeles County Museum of Natural History (**LACoMNH**), Scripps Institute of Oceanography (**SIO**), the Monterey Bay Aquarium Research Institute (**MBARI**), Moss Landing Marine Laboratories (**MLML**) and the small museum which is a part of the Cabrillo Marine Aquarium in San Pedro, California (**CMA**) (see Appendix 1: List of material examined). These were compared to SBMNH specimens, to identify species represented in the collection. Additionally, specimens collected by the Olympic Coast National Marine Sanctuary (OCNMS) on several expeditions (2006, 2008) were examined, as were specimens from various National Oceanographic and Atmospheric Administration (NOAA) offices from both the western and eastern United States. However, extensive collecting of gorgonians was not generally required for this collection reorganization and review; in future, collecting of further specimens may be needed.

Any new material that may have come in was supplied through standard collection procedures that are employed by others (NOAA, LACSD, OCSD) in their on-going, survey-based collection events. A succinct yet comprehensive overview of collection procedures can be found in Etnoyer et al. (2006). The initial part of the study required that material in the collection be checked for correct storage conditions; if they were not so stored, preparation for long-term storage was undertaken.

Approximately two-thirds of the SBMNH collection is stored wet, in 70% ethanol; the other third of the collection is dry stored. When the cnidarian material from AHF came into the SBMNH’s possession, all of the wet material had to be properly stored, then original hand-written labels with collection data had to be preserved for both wet and dry specimens and the museum’s official institutional labels had to be printed and stored with the original label. All specimens discussed in this publication, housed at SBMNH, were cataloged as voucher specimens and data for these specimens were entered into the Museum’s online database.

All colonies were examined for gross morphology; records of height and width of a colony, length of main and secondary branches, diameter of those branches, color of the axis (if visible), and color of the colony were made. No molecular taxonomic work was undertaken with specimens received from AHF. As no formalin should come in contact with the samples, and there was no certainty that any of the material in the SBMNH collection had not encountered formalin, molecular extraction was not considered. A fuller discussion on methods for preserving and handling tissue for molecular study (DNA or RNA analysis) can be found in Etnoyer et al. (2006).

For this review, examination of the calcareous sclerites, present in different parts of the colony, was conducted for nearly all specimens. Two specimens lying side by side, and appearing similar in color and overall colony form could have very different sclerites, thus establishing them as potentially different species (however, as sclerites themselves can be environmentally “plastic,” which can complicate species identification). This was a common theme regarding certain genera present in the California Bight as reflected in the SBMNH collection. Geographic location of the specimen or sample collected, as well as details/specifics (depth, degree of current flow, etc.), aided in identifications. Following sclerite preparation, using the standard method for sclerite extraction (tissue sample in common household bleach), light microscopy via a compound Olympus (CH) microscope, was used initially to determine the genus to which a specimen belonged; careful examination of sclerite details then allowed for identification to species. Sclerites were then photographed using a digital camera system (Olympus BH2 microscope with an Olympus Q Color 5 digital camera) able to record a millimeter scale on each image. Scanning Electron Microscopy (SEM) of the sclerites was then undertaken. All samples were coated with gold, using a Cressington Sputter Coater Unit, 108auto. Samples were examined and digital images taken, using a Zeiss Scanning Electron Microscope EVO 40, at 10 kV. Sclerites were chosen from stub arrays that best displayed the key features of variety, size, shape, or ornamentation; despite bits of organic debris or some damage, if a sclerite, overall, was a good representation of a “key” sclerite shape, it was used. Images were both stored on computer hard-drive and cataloged on external drives, for permanent, backup archival storage. SEM stubs were housed in plastic stub boxes; stubs were numbered with Museum Catalog numbers and each box bears a paper label, listing all species with their museum number, contained in that particular box. This SEM information was then entered into the Museum’s on-line database, supplementing all other key information known for each specimen. SEM was used to confirm identification in all species, through comparison against SEM images found in published literature of species with verified identification. In the end, some 40-plus species of gorgonian, from approximately 23 genera, were identified, cataloged, and appropriately housed. This first part covers a dozen species, those classified as scleraxonians as well as those from the family Acanthogorgiidae (Holaxonia). A summative overview of species housed in the SBMNH research collection, from these specific groups, is included above. As further exploratory work is undertaken in the CA Bight, and adjacent areas, likely more species will be added to the list, and (hopefully), new specimens will find their way into the SBMNH research collection.

This information regarding species and lots of specimens, examined for Part I, both for colony morphology and sclerites (either through light microscopy or SEM) is a summation of the more detailed information to be found in the Appendix 1: List of material examined – Part I. It is evident from this summative overview that the SBMNH research collection (while illustrative of species considered part of the scleraxonian group or the holaxonian Acanthogorgiidae found within or near the California Bight) is not extensive, nor likely fully representative of all species of scleraxonian and Acanthogorgiidae that could potentially exist in the California Bight region.

**Table:** Part I: Collective specimen and species data.

# of specimens analyzed with sclerite preparations	32
# of specimens examined without sclerite preparation	1
Breakdown of specimens examined:	
# of specimens analyzed from SBMNH collection	13
# of specimens analyzed from USNM-Smithsonian	13
# of specimens analyzed from CAS	2
# of specimens analyzed from other institutions	4
Total # of species that received sclerite observations	12
# of new species described	1
Breakdown of species examined:	
# of species from the SBMNH collection	6
# of species from USNM-Smithsonian	11
# of species from CAS	1
# of species from other sources	4
# of species shown in Figures (colony)	5
# of species shown in Figures (either light microscopy and/or SEM of sclerites)	4

**Table:** Species covered in this part.

	**SBMNH**	**Other institutions**	**Colony figure**	**Sclerite figure**
* Anthothela pacifica *	Yes	Yes	Yes	Yes
* Paragorgia arborea *	Yes	Yes	Yes	No
* Paragorgia regalis *	No	Yes	No	No
* Paragorgia stephencairnsi *	No	Yes	No	No
* Paragorgia yutlinux *	No	Yes	No	No
*Sibogagorgiacalifornica* sp. nov.	Yes	Yes	Yes	Yes
* Hemicorallium ducale *	Yes	Yes	No	No
* Hemicorallium imperiale *	No	Yes	No	No
* Hemicorallium regale *	No	Yes	No	No
* Acanthogorgia gracillima *	Yes	?	Yes	Yes
*Acanthogorgia* sp. A	Yes	?	Yes	Yes
* Muricella complanata *	No	?	No	No

## Systematic accounts

(Classification used throughout this paper conforms to that of Bayer 1981c)

### Diagnosis of the order Alcyonacea Lamouroux, 1816

(Gorgonian corals, as defined previously)

Octocorals with uniformly short gastrovascular cavities; colonies typically arborescent, rarely lobate or incrusting, producing more or less specialized three-dimensional axial skeletal structures: either a distinct central axis of horny (gorgonin) or calcareous material (or both), or a central medullar zone of calcareous sclerites which are loosely or inseparably bound together by horny or calcareous matter.

#### Scleraxonia Studer, 1887

Octocorals with central axis, medullar zone or inner layer containing sclerites bound together more or less solidly either by horny or calcareous material; outer layer of coenenchyme containing proximal portions of gastrovascular chambers of polyps, endodermal canals and solenia; cortical sclerites free, commonly appearing different from those in medullar region; axial cylinder/medulla may contain canals and solenia but polyp cavities do not penetrate; cross-chambered central chord absent; sclerites always present.

##### Key to Families represented in SBMNH collection (Scleraxonia)

**Table:** 

1	Axis with spongy, horny nodes, filled with sclerites alternating with longer calcareous internodes composed of cemented sclerites	**(not discussed in this publication)**
–	Axis not composed of alternating horny nodes and calcareous internodes	**2**
2	Axis a medullar region composed of completely fused calcareous sclerites forming a central cylinder of solid calcium carbonate	**Family Coralliidae**
–	Axis a medullar region composed of multiple, separate, sclerites held together by a horny material	**3**
3	Axis with a cross-chambered central chord	**(not discussed in this publication)**
–	Axis without a chambered central chord, but often with numerous gastrodermal canals	**4**
4	Cortex set off from medulla by a ring of boundary canals; polyps monomorphic and protruding	**Family Anthothelidae**
–	Cortex not set off from medulla by a ring of boundary canals; medulla formed only by unfused sclerites, penetrated throughout by longitudinal canals; polyps retractile, not protruding	**Family Paragorgiidae**


##### List of species of Scleraxonia Studer, 1887

Class Anthozoa

Subclass Octocorallia Haeckel, 1866

Order Alcyonacea Lamouroux, 1816

**Suborder Scleraxonia Studer, 1887**

Family Anthothelidae Broch, 1916

*Anthothelapacifica* (Kükenthal, 1913)

Family Paragorgiidae Kükenthal, 1916

Paragorgiaarboreavar.pacifica (Verrill, 1922)

*Paragorgiaregalis* Nutting, 1912

*Paragorgiastephencairnsi* Sánchez, 2005

*Paragorgiayutlinux* Sánchez, 2005

*Sibogagorgiacalifornica* sp. nov.

Family Coralliidae Lamouroux, 1812

*Hemicoralliumducale* (Bayer, 1955)

*Hemicoralliumimperiale* (Bayer, 1955)

*Hemicoralliumregale* (Bayer, 1956)

##### Descriptions of species of Scleraxonia Studer, 1887

###### Family Anthothelidae

Taxon classificationAnimaliaAlcyonaceaAnthothelidae

Broch, 1916

####### Diagnosis.

Branches of colonies slender. Polyps monomorphic, with prominent calyces, anthocodiae usually exsert. Axis not jointed, without a cross-chambered central core. Medulla surrounded by longitudinal boundary canals (of roughly equal size) separating it from cortex; medulla only rarely perforated by gastrodermal solenia in smaller branches and even then, not as extensively as in lower parts of colony; in larger branches, medulla perforated by solenia. Generally, medulla with separable sclerites; medullar sclerites stout spindles (not needle-like), thorny, ornamented with warts, spines or branching processes, that may link sclerites together. Sclerites of coenenchyme longer fusiform spindles, sometimes clavate or bent, occasionally with radiate bodies and capstans (rarely).

####### Discussion.

Within the Subclass Octocorallia, taxonomic placement of this family reflects the changeable history the Order Alcyonacea has experienced since its inception. Currently, Alcyonacea is one of three orders in the subclass (Williams and Cairns 2014). The current Order Alcyonacea was, however, originally divided into four orders (Alcyonacea, Gorgonacea, Stolonifera, and Telestacea). Current coral taxonomy now divides Order Alcyonacea into five nominal groups: Calcaxonia, Holaxonia, Scleraxonia, Alcyoniina and Stolonifera (Bayer 1981c, Fabricius and Alderslade 2001). While the Family Anthothelidae is today recognized as valid in the Order Alcyonacea [Scleraxonia], a number of species in the family were originally placed in the older Order Stolonifera, often within the Family Clavulariidae. A few researchers may still group some of the families of soft corals in an Order Stolonifera, but since then, a number of genera and several species have been moved out of Stolonifera. Fabricius and Alderslade (2001) noted that the “decision whether to categorize a particular genus as a stoloniferan becomes so subjective that the name plainly has limited classificatory value . . . . .” Use of the Order Stolonifera, and placement of the Family Anthothelidae in it, or the nominal group, Scleraxonia (as opposed to Stolonifera), has had a continued, tumultuous history (Hickson 1915, Molander 1918, Kükenthal 1919, 1924, Madsen 1944, Bayer 1961, Bayer 1981a, b, c, Hochberg 1979). Currently, the classification of species in the family is determined by the presence of a membranous colony form, presence, or absence of coenenchyme layers surrounding an axis, and the way in which polyps arise from the membranous base.

Any membranous octocoral colony currently held in the SBMNH collection (few in number, small in size, deteriorated due to early formalin storage) could be a member of either the genus *Clavularia* or the genus *Anthothela* (the latter, a genus within the Order Alcyonacea, Scleraxonia). A detailed examination of the few colonies held in the SBMNH collection, in comparison with material housed elsewhere is needed; that necessitates a separate project, to be undertaken at some future date. Most of the material in the SBMNH collection with membranous colony form is present in a very fragile state; a more detailed description for each will not be easy, in some instances, not possible at all. It is likely that even with a more thorough examination of the material held at SBMNH, the results will necessarily be inconclusive. A complete revision of the genus *Anthothela* was recently completed by Moore et al. (2017), utilized here.

###### Genus Anthothela

Taxon classificationAnimaliaAlcyonaceaAnthothelidae

Verrill, 1879

Briareum Sars, 1856b: 63 [pars].
? Gymnosarca Kent, 1870a: 397. Stephens, 1909: 7.
Anthothela Verrill, 1879a: 199; 1883: 40. Studer (and Wright) 1887: 28. Grieg 1891: 3. Broch 1913: 4. Kükenthal 1919: 43; 1924: 14. Thomson and Dean 1931: ? 11–20. Stiasny 1937: 20. Verseveldt 1940: 37. Bayer 1961: 57–58 (key), 65 (key), 67–68, 70. Arantes and de Medeiros 2006: 2. Moore et al. 2017: 19.
? Stereogorgia Kükenthal, 1916: 178.
Suberia Nutting, 1911: 15.

####### Type species.

*Briareumgrandiflorum* Sars, 1856b (by subsequent designation).

####### Diagnosis.

With slender, rounded, tortuous, commonly upright, abundant anastomosing branches producing tangled colonies. Branches always solid; no main stems developed, branches grading upwards from broadened membranous base. Polyps present on base as well as on branches, widely scattered on all sides, sometimes clustered into large masses. Polyps elongated in expansion arising from distinctly projecting, short yet elevated cylindrical calyces. Polyps partially retractile, seldom entirely retractile; large anthocodiae commonly preserved exsert, arising from either extended, rather thin, slightly sharp, spiculose, but spongy, basal membrane (encrusting) or from slender irregular stems (branched). Axis spiculose, well differentiated, not firm. Long, strongly warted, often irregular spindles and short, girdled rods in coenenchyme. Sclerites of axis more irregular; bear fewer, larger warts, knobs or lobes. Spongy base filled with thin spindles and rods, permeating tissue. Bright rosy-red or brownish in life, but other color forms likely.

###### Anthothela pacifica

Taxon classificationAnimaliaAlcyonaceaAnthothelidae

(Kükenthal, 1913)

Figures 1A, B , 2

Anthothela pacifica (Kükenthal, 1913a): 237–239; text figs E–G. Bayer 1961: 336. Moore et al. 2017: 34–40.
(?) Clavulariapacifica Nutting, 1909: 686. Kükenthal 1913a: 237–239. Hickson 1915: 548.
(?) Sympodiumarmatum Wright & Studer, 1889: 272. Nutting 1909: 686.
(?) Anthothelaargentea Studer, 1894: 60. Kükenthal 1919: 45; 1924: 16. (No longer valid; see WoRMS Data Base, Cordeiro et al. 2018a, Anthothela)

####### Type locality.

USA, California, China Point, (?) San Clemente Island, SW tip, 50 fm (91 m).

####### Type specimen.

Location of type unknown.

####### Material examined.

One specimen in SBMNH collection was identified as this species (see Appendix 1: List of material examined).

####### Description.

**Figure 1.**
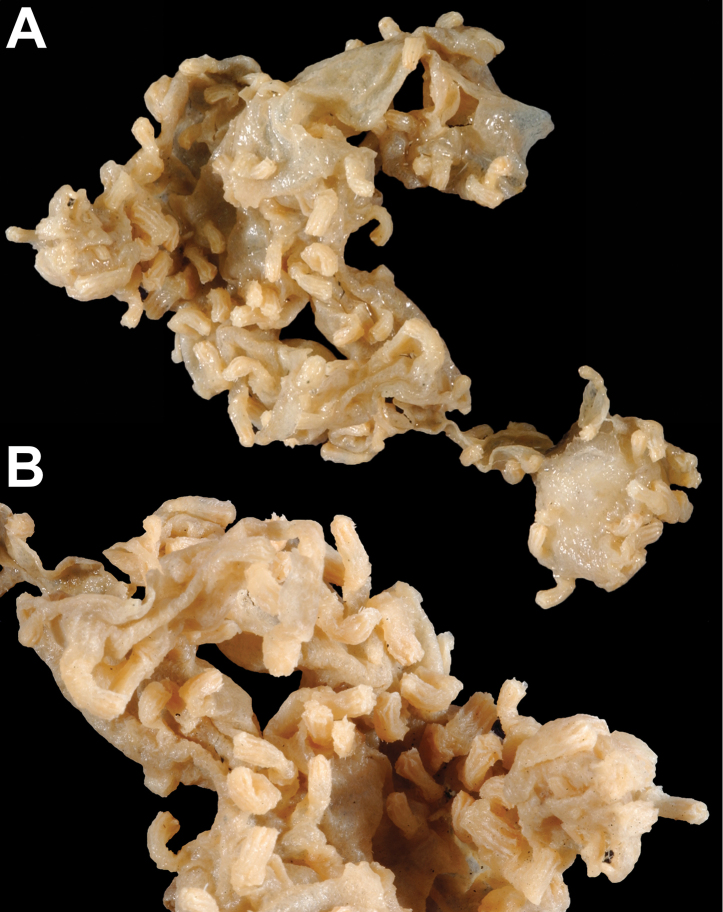
*Anthothelapacifica*, SBMNH 265939. **A** Large portion of colony fragment; several rocks accompanied specimen (presumed substrate); colony unattached, ~7.0 cm in length **B** close-up of polyps, each polyp ~0.5 mm tall.

**Figure 2.**
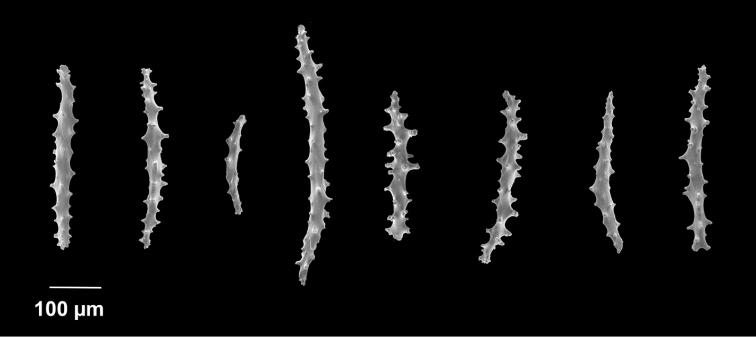
*Anthothelapacifica*, SBMNH 265939, SEM image. Coenenchymal sclerites, colored a very pale yellow to white. Length of largest sclerites examined ~400 µm.

*Colony* (Figure 1A) of thin, flattened branches, crooked, tortuous, somewhat anastomosed; no large main stem development. Rather low-lying, prostrate form; height 0.5 mm-7.0 mm; “stolon” thin, membranous; arrangement of medulla and “cortex” as described for family. Calyces (Figure 1B) prominent, projecting cylinders up to 2.5–3.0 mm tall; polyps stout, with highly retractile portion up to 2.5 mm in length; total calyx/polyp size 5.0 mm tall by 2.0 mm wide. Calyces monomorphic, with eight deep, longitudinal grooves, delineating eight ridges or ribs with sharp edges. Distal part of polyp usually exsert, bearing eight double-rows of spindles. Polyps large, rather widely spaced. Color of living colony described as light yellow or straw-colored to grayish white; in alcohol, creamy white. Sclerites (Figure 2) of coenenchyme large, elongate, thin, pointed spindles, often with prominent projections on edges; generally, fusiform (0.25–0.3 mm long), not capstans; often appearing bent. Older descriptions indicate that sometimes these are strongly clavate at terminal end, appearing as clubbed spindles; this condition not seen in material examined. Anthocodial armature strong spindles, often clavate or bent, only rarely as radiate bodies and capstans. Spindles at base of tentacles (collaret), 0.18 mm long, those of calyx 0.22 mm long, none of these club-shaped. Sclerites widely spaced, showing transverse disposition at base of tentacles; sclerites of medulla strong thorny spindles. Colony surface rough to the touch due to projecting sclerites.

####### Etymology.

Presumably named for type locality, northern Pacific Ocean.

####### Common Name.

Dwarf white gorgonian.

####### Distribution.

Not definitively known at this time for coastal western United States; potentially from southern California: USA, California, San Diego, Point Loma, 200 m (Nutting 1909) and China Point, (?) San Clemente Island, SW tip, 91 m, to at least northern California, Monterey Bay, 900 m (Nutting 1909); possibly as far north as southern British Columbia, Canada (Lamb and Hanby 2005).

####### Biology.

According to Kozloff (1987), a shallow subtidal form. Lamb and Hanby (2005) state it as “subtidal, below 40 m (133 ft).”

####### Remarks.

Kükenthal (1913a) indicated species is not equal to *Sympodiumarmatum* Wright and Studer as cited in Nutting (1909). However, an interesting aspect presented itself through an unexpected source. A letter, found in SBMNH archives, dated 1978, from Dr Frederick Bayer to Dr Eric Hochberg, indicated the suggestion of a possible synonymy between *Anthothelaargentea* Studer, 1894 and *Anthothelapacifica*. In that letter Dr Bayer made two significant statements, the first being that the two lots reported and identified (and misidentified) by Nutting (1909: 686) as “*Sympodiumarmatum* Wright & Studer, 1889,” subsequently identified by Kükenthal (1913) as *Clavulariapacifica*, are in actuality gorgonians of the genus *Anthothela*. Bayer (1961) made mention of this in relation to the Atlantic Ocean species *Anthothelatropicalis* Bayer, 1961. Secondly (key point of the letter), Bayer believed that it was “possible that ‘Clavularia’ pacifica is identical with *Anthothelaargentea* Studer, in which case Studer’s name takes precedence.” Further reading in Bayer (1961) showed that there was indeed a genus name change. Bayer, discussing family Anthothelidae, genus *Anthothela*, made the following statement: “A distinctly different species has now been recorded from the Gulf of Mexico, also in deep water, which proves to be a new species closely related to the eastern Pacific *Anthothelapacifica* (Kükenthal), forming with it an eastern Pacific-western Atlantic twin-pair of species.” Note the change in genus name (from *Clavularia* to *Anthothela*). Regarding the distributional range for *A.argentea*, location records noted on distribution maps posted on the Encyclopedia Of Life website showed it occurring throughout the southeastern Pacific Ocean, extending into the northeastern Pacific, to at least central California (USA; USNM 94428 was collected in proximity of the western edge of the Bight). This indicated a possible geographic overlap with (or the possibility that it was synonymous with) *A.pacifica*. However, the work of Moore et al. (2017) puts the suggested synonymy of Bayer in question, having reassigned *Anthothelaargentea* to the genus *Victorgorgia* López-González & Briand, 2002 and the new family Victorgorgiidae, as they indicated that there are clear morphological and genetic differences from the genus *Anthothela*.

From the World List of Octocorallia, the World Register of Marine Species (WoRMS), *Anthothelapacifica* is an accepted scientific name, while *Anthothelaargentea* has been accepted as *Victorgorgiaargentea*, and from that listing it is clear that these two are considered separate species (Cordeiro et al. 2018a).

Identification of specimen relied on notes made by Dr Hochberg, with a description given by Kükenthal; fragility of specimen did not permit an extensive examination, but as far as it could be done, one was done with the specimen to hand.

Hickson (1915) lamented: the “widely distributed and very variable genus *Clavularia* is badly in need of revision. It is probable that such a revision would lead to a considerable reduction in the numbers of species, many of which have been founded on very in-adequate characters.” He went on to say that the genus *Clavularia* is well represented in the North Pacific Ocean; how many other species belong in the genus *Clavularia*, and how many may be members of the genus *Anthothela*, or some other genus, remains to be seen. Bayer (1961:70) affirmed that Nutting’s and Kükenthal’s specimens are gorgonians of the genus *Anthothela*. Hochberg (1979) stated, “comparison with the type is needed to identify what has been called *Clavulariapacifica* from this area in the past. The generic change (to *Anthothela*) is an obscure reference, being only a few lines in Bayer’s work (1961) on Caribbean octocorals.”

Location of type is at issue; someone (unknown) noted: “China Point, ‘San Clemente Island (SW tip).’ ” There was no way to confirm this statement, and there is the added problem of a China Point on Santa Catalina Island (SW side of island), as well. There was no means to identify which “China Point” was the correct collection location.

###### Family Paragorgiidae

Taxon classificationAnimaliaAlcyonaceaParagorgiidae

Kükenthal, 1916

####### Diagnosis.

Robust, profusely branched colonies with dimorphic polyps (feeding autozooids, reproductive siphonozooids). Axial skeletal structure solely a continuous medulla, containing separable sclerites. Medulla perforated by gastrodermal canals all the way to branch tips, not separated from cortex by a ring of boundary canals.

###### Genus Paragorgia

Taxon classificationAnimaliaAlcyonaceaParagorgiidae

Milne Edwards & Haime, 1857

Paragorgia Milne Edwards & Haime, 1857: 190. Verrill 1878b: 476. Kükenthal 1919: 77 [synonymy]; 1924: 28 [synonymy]. Verseveldt 1940: 137. Bayer 1956a: F197; 1993: 2. Sánchez 2005: 15.

####### Type species.

*Alcyoniumarboreum* Linné, 1758; [= by subsequent designation, *Paragorgiaarborea* (Linnaeus, 1758), by monotypy].

####### Diagnosis.

Massive, tree-like colonies with thick branches, measuring up to 7.0 meters tall, perhaps as much as 6.0–7.0 meters wide. Sclerites in axial medulla, long, ornate rods (spindles) with branching processes, derived from capstan type, up to 0.6–0.8 mm in length, colorless or pink; elsewhere (coenenchyme, tentacles, etc.) sclerites small (less than 0.15 mm in length), differing shapes, commonly pink or red. Surface sclerites six-, seven-, and eight-radiate capstans, always less than 0.1 mm long, with globular, smooth, grooved or lobulated ornamentation. Sclerites in subsurface/outer medulla of intermediate form, ranging between radiates and spindles. Autozooid polyp tentacles have distinctively blunt, stubby rods or ovals, less than 0.1 mm.

###### Paragorgia arborea var. pacifica

Taxon classificationAnimaliaAlcyonaceaParagorgiidae

(Verrill, 1922)

Figure 3

**Figure 3.**
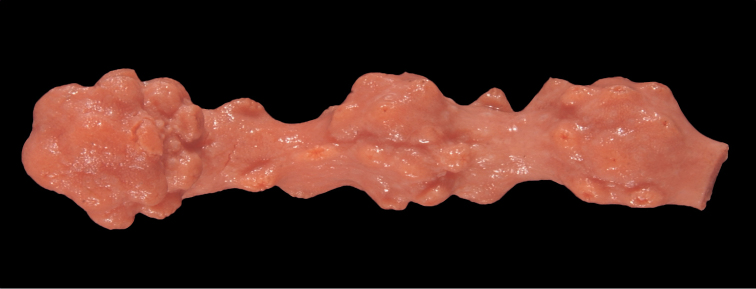
Paragorgiaarboreavar.pacifica, SBMNH 422977. Single branch fragment, ~5.0 cm long, displaying knobby condition of branches seen in some specimens.

**Figure 4.**
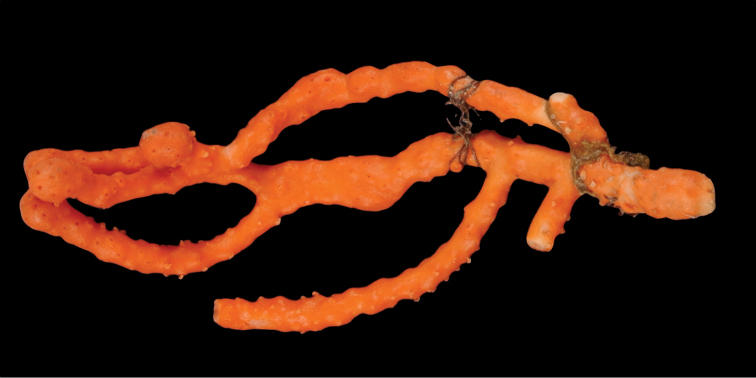
*Sibogagorgiacalifornica* sp. nov., SBMNH 422974 (Holotype). Large fragment, ~18 cm long, showing branching pattern; intertwined tendrils from shark egg case are visible.

Paragorgia pacifica (Verrill, 1922): G16–G18; plate VIII, figs 3, 4b.
Alcyonium arboreum Linnaeus, 1758: 803. Pallas, 1787: 164.
Paragorgia arborea (Linnaeus, 1758): 803. Milne Edwards 1857: 190. Broch 1912: 6. Hickson 1915: 548–549. Grasshoff 1979: 117 [and references therein]. Sánchez 2005: 15–20.
Paragorgia nodosa Koren & Danielsson, 1883: 19 [sensu Bayer 1956b: 70].
(?) Paragorgianodosa Nutting, 1912: 99.
(?) Paragorgiaregalis Nutting, 1912: 100.

####### Type locality.

Canada, British Columbia, Vancouver Island, Jervis Inlet, ~20 m.

####### Type specimens.

**Holotype** YPM-5373 [dry].

####### Material examined.

~1–2 lots (see Appendix 1: List of material examined).

####### Description.

Collection lot studied contains one branch fragment (Figure 3); in most respects, examination of fragment revealed characters that align with the description given in Sánchez (2005, pages 15–20). The branch is distinctive in its knobby aspect, but sclerites fall well within the parameters of morphology as discussed and shown in Sánchez (2005).

####### Etymology.

The variety name *pacifica* was presumably proposed in reference to the location/distribution of the species in the Pacific Ocean.

####### Common name.

Referred to frequently as “Bubblegum coral.” Cairns et al. (2003) referred to Breeze et al. (1997) where this genus/species is also listed as “Rubber Trees” and “Strawberry Plants.” Specifically, it could be called the “Pacific bubblegum tree.”

####### Distribution.

Recorded from Alaskan waters, Bering Sea, ‘Albatross’, 54°02'40"N, 166°42'00"W, at a depth of 504 m; USNM 3315. Also, recorded from Unalaska to Kodiak, ‘Albatross’, 54°19'00"N, 159°40'00"W, taken by dredge, 114 m; USNM 3338. Bayer indicated (unpublished ms 2, Cairns 2009) that known distribution of this species was from the Bering Sea south to Vancouver Island (specimen collected by Mr Wm Spreadborough, at Ucluelet, Vancouver Island, BC, at a depth of 16 m, June, 1909. [Col. No. 51, Coelenterates, Victoria Memorial Museum, Ottawa]) on the east side of the Pacific, and likely over to Japan on the west; southern limit (at the time Bayer was writing) was unknown. Based on more recent work, including the review of systematics for the family by Sánchez (2005), this species, in the Pacific Ocean (a possible variant of *P.arborea*), extends to at least the northern limit of the California Bight on the eastern side (at question, further south, at depth), and down into New Zealand waters on the Pacific Ocean’s western side.

####### Biology.

Specimens collected or photographed in the Monterey Bay Marine Sanctuary have harbored polychaete worms, purple in color (species identification not determined), with the worms wound around the branches of the colony (Langstroth and Langstroth 2000). On a specimen examined from Mexico, Gulf of California, Baja, Bahia de Los Angeles, there was a complete over-covering of what may be some sort of grey colonial or zoanthid-type organism (the specimen was not included in the list of material examined, as the overgrowth of the zoanthids precluded any clear examination of the host gorgonian itself). A MBARI video clip, viewed on a visit to MBARI, had an excellent segment of this species heavily colonized by numerous basket stars, so many in fact, that the entire, large tree-like colony displayed a heavy growth of “hair.” According to Langstroth and Langstroth (2000), on a *Paragorgia* specimen, a feather star, *Florometraserratissima*, was seen clutching the gorgonian with its leg-like cirri. While this was seen in a lab setting, the feather star likely may have come with the gorgonian during the collection process. If so, other filter-feeding echinoderms might be seen living on/with these gorgonians in situ. Evidence from recent OCNMS expeditions, as well as numerous MBARI and NOAA video clips support this. Colonies living in deeper water grow very slowly in some areas and could be several hundred years old (Andrews et al. 2005, Sherwood et al. 2005), reaching heights of several meters (DeVogelaere et al. 2005). It is speculated (Brancato et al. 2007) that these large, aged colonies provide critical habitat for such organisms as Northern Rockfish, Pacific Ocean Perch, species of King Crab, and Pacific Cod. An expedition undertaken by Olympic Coast NMS (May 2006) lent credence to this speculation.

####### Remarks.

Sclerite examination of the sample shown in Figure 3 agreed with those seen in Sánchez (2005: fig 9); the six-radiates distinctive of this species were confirmed. Verrill (1922), reporting on specimens of *Paragorgiaarborea*, discussed the possibility of the existence of this variant. Bayer stated in his unpublished manuscript (ms 2, Cairns 2009)) that “*P.arborea* seems limited to latitudes of 40° or higher . . . . *P.arborea* seems to be truly bipolar, since no reliable finds have been made south of British Columbia in the Pacific. It is impossible to be sure that the species does not show equatorial submergence.” Thus, “it appears that the genus *Paragorgia*, an inhabitant of cold waters, . . . whose various species occur at moderate depths in boreal and anti-boreal regions, follow the cool water to greater depths in low latitudes.” Bayer additionally stated that a specimen obtained in British Columbia, as *P.pacifica* could be a variety of *P.arborea*. Kozloff et al. (1987) stated that *Paragorgiapacifica* is “the most commonly encountered gorgonian of” the Pacific Northwest region; “it has been called *Paragorgiaarborea* Linnaeus, 1758.” The final word comes from Sánchez (2005); he stated that a comprehensive review of North Pacific populations of *P.arborea*, “including type material and genetics is needed before reaching conclusions on *P.pacifica* and the differentiation of south vs. north *P.arborea* populations.”

The California Academy of Sciences has approximately 30 lots of this, or other species, attributed to this genus; most specimens are from Alaska; as well, two are from the USSR, one is from Norway, and one is from Oregon. Nine of the remaining lots are specimens collected from California; most are from Monterey Bay, with one from the Davidson Seamount. Of these nine, only three have been identified as being this particular species. MBARI has extensive video records of this species from Monterey Canyon, as well as the Davidson Seamount. In the Moss Landing Marine Labs collection there is a small specimen of what may well be this species, collected in Monterey Bay, ~36°27'12"N, 122°04'02"W, ~450 meters; coll. G McDonald, 13 March 1974; C0067 [wet]. As well, there is an impressive, tree-sized dry specimen on display in the hallway near the museum door. (Collection data may be available for this specimen, but collection data could not be located.)

Based on multiple examinations of possible *Paragorgia* material in the SBMNH research collection, none (one exception) were examples of P.arboreavar.pacifica; numerous sclerite preparations never revealed the six-radiate sclerite form that is characteristic of this species; only a very few displayed eight-radiates. As well, no polyp sclerites were ever obtained. As most of the SBMNH specimens examined clearly lacked the “key” identifying sclerites, no request was made to obtain the holotype specimen from Yale Peabody Museum. *P.arborea* (though not the variety *pacifica* suggested by Verrill) is an accepted species in the WoRMS listings (Cordeiro et al. 2018b). As well, the three following species are also accepted species in the WoRMS registry. These are included as they have been collected in very close proximity to the California Bight region. None however, are represented in the SBMNH collection and research indicated that they were never collected on any of the ‘Velero’ expeditions. In point of fact, there were no records of any specimens in the genus *Paragorgia* having been collected.

###### Paragorgia regalis

Taxon classificationAnimaliaAlcyonaceaParagorgiidae

Nutting, 1912

Paragorgia regalis Nutting, 1912: 100. Sánchez 2005: 25–29.
Paragorgia dendroides Bayer, 1956b: 69 [sensu Bayer 1964a: 526]. Grasshoff 1979: 120.

####### Material examined.

No material in SBMNH collection (see Appendix 1: List of material examined).

####### Remarks.

Inclusion of *P.regalis* is based on USNM 1027063, which was collected just west of the California Channel Islands, in proximity to western boundary edge of Bight. Moss Landing Marine Labs may have a representative sample of this species in their collection, from Monterey Bay Canyon, 36°25'54"N, 122°00'03"W, ~900 m; coll. D Rold and H King, December 1978; C0074 [wet]. It is possible that some of the *Paragorgia* seen in MBARI video segments could be this species; this species may have been seen when examination of samples of gorgonian collected by J Barry (MBARI) was done, made available for examination by Lonny Lundsten. Distribution ranges across the Pacific Ocean (based on collection records for specimens in NMNH). Collection depths in Hawaii and Japan range from 452–1840 m (Sánchez 2005), and as noted from NMNH collection. For a common name, this species might be called the “Regal bubblegum tree.”

###### Paragorgia stephencairnsi

Taxon classificationAnimaliaAlcyonaceaParagorgiidae

Sánchez, 2005

Paragorgia stephencairnsi Sánchez, 2005: 57–60; figs 39–41.

####### Material examined.

No material in SBMNH collection (see Appendix 1: List of material examined).

####### Remarks.

A paratype specimen at NMNH (USNM 94437), identified as this species, was collected in proximity of the southern California Channel Islands, in an area lying close to the western boundary of the California Bight; range seems to extend northward to an area off British Columbia, Canada (location data for USNM 57982, the holotype; discussed in Sánchez 2005). As this species was named in honor of Dr Stephen Cairns of the National Museum of Natural History, Smithsonian, it would be fitting to call this “Stephen’s bubblegum tree.” Any similarities between this and other species in the genus were thoroughly discussed in Sánchez (2005).

###### Paragorgia yutlinux

Taxon classificationAnimaliaAlcyonaceaParagorgiidae

Sánchez, 2005

Paragorgia yutlinux Sánchez, 2005: 53–57; figs 36–38.

####### Material examined.

No material in SBMNH collection (see Appendix 1: List of material examined).

####### Remarks.

A specimen at NMNH (USNM 90345), identified as this species, was collected west of the southern California Channel Islands in an area that lies near the western boundary of the California Bight. A MBARI sample taken from Monterey Canyon, near Point Sur, on 7 April 2008 could also be this species. A number of other samples, examined cursorily, housed at MBARI, could be this species.

###### Genus Sibogagorgia

Taxon classificationAnimaliaAlcyonaceaParagorgiidae

Stiasny, 1937

Sibogagorgia Stiasny, 1937b: 80. Sánchez 2005: 60. Herrera et al. 2010: 132.

####### Type species.

*Sibogagorgiaweberi* Stiasny, 1937b: 80 [= *Suberia köllikeri* Nutting, 1911: 13, nec Studer 1879].

####### Diagnosis.

A genus in the family Paragorgiidae with scleritic medulla showing no (or very few, one to two) large penetration canals; main solenia around subsurface/outer medulla as boundary canals, forming reticulate network; network of canals observable with light microscopy as a regularly reticulate and uniform mesh just beneath surface. Polyps without tentacular sclerites, outer surface with radiate sclerites; generally, medullary sclerites nearly bare of ornamentation. Autozooid polyps uniformly to randomly distributed along branches, throughout colony. Branching colonies often in one plane (but not always); main branches usually thicker than terminals; terminals clavate. Coloration either of uniform beige or bright orange-red to a beige coloring with slightly projected pinkish orange polyp apertures.

###### Sibogagorgia californica sp. nov.

Taxon classificationAnimaliaAlcyonaceaParagorgiidae

http://zoobank.org/5B485BEF-92D9-4A35-923F-BC491076AC72

**Figure 5.**
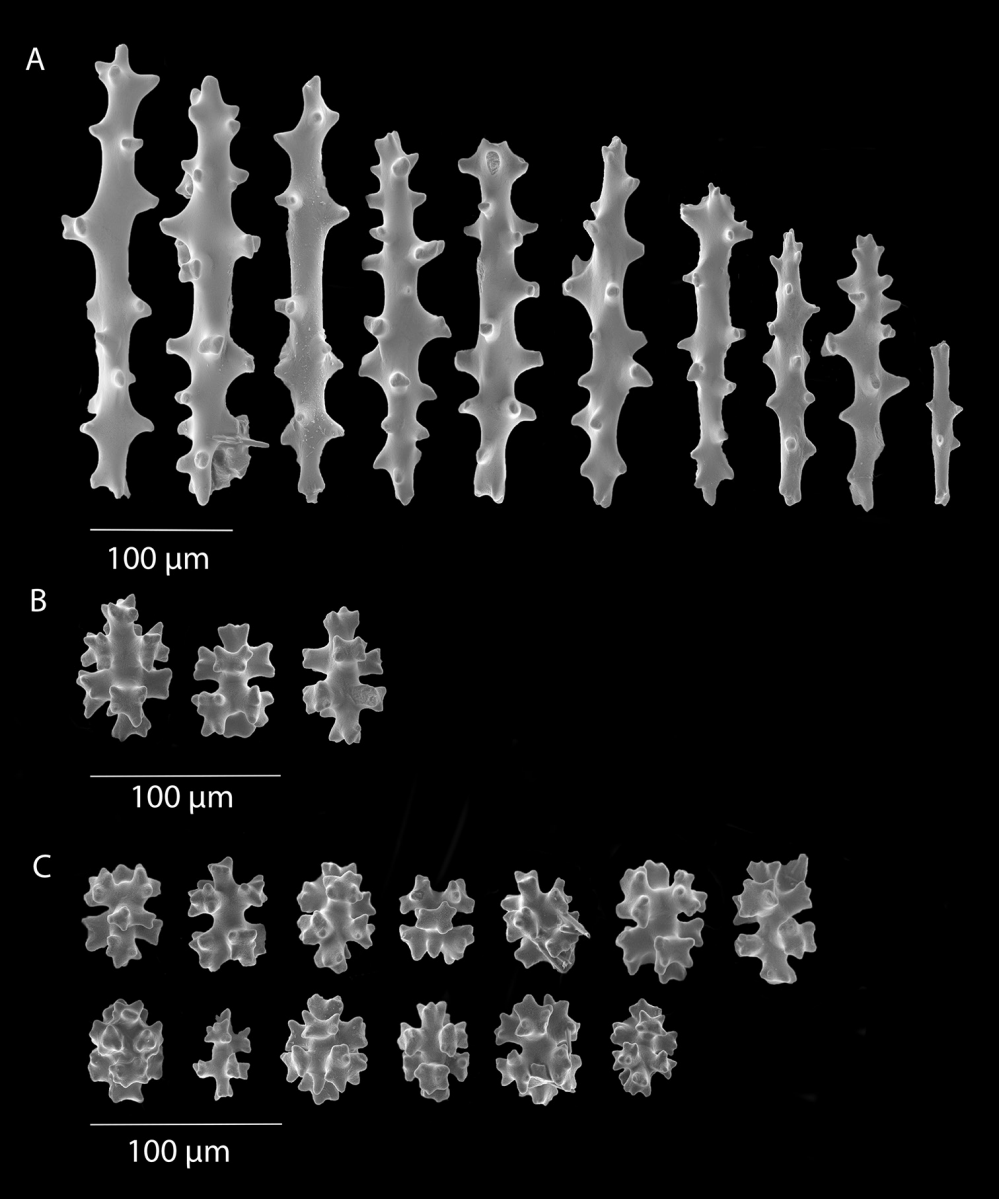
*Sibogagorgiacalifornica* sp. nov., SBMNH 422974, SEM image. Sclerites either bright salmon in color, or pale yellow to white. **A** Sclerites from colony medulla **B** sclerites from colony surface and possible intermediates toward the medulla **C** 7-radiate sclerites from colony surface.

**Figure 6.**
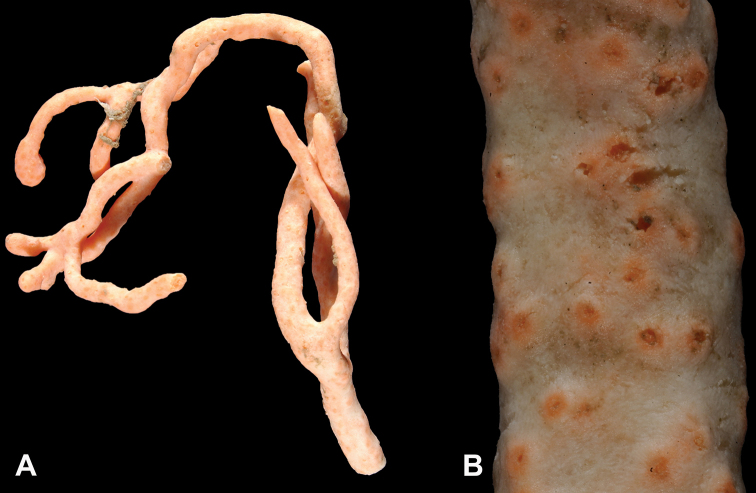
*Sibogagorgiacalifornica* sp. nov., SBMNH 422978. **A** Large, ~36 cm long colony fragment **B** close-up view of polyps, illustrating color and position on branch; note contrast color between polyps and surrounding coenenchyme, characteristic of species in the genus. This may however, be a partially bleached specimen, rather than one exhibiting true coloring.

**Figure 7.**
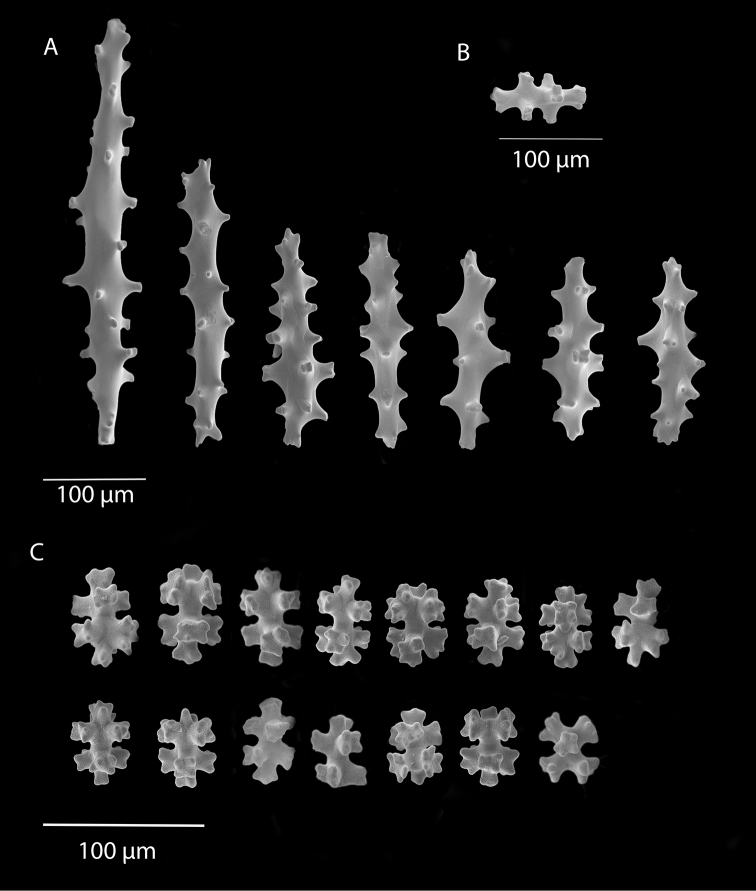
*Sibogagorgiacalifornica* sp. nov., SBMNH 422978, SEM image. Color of sclerites from this colony very pale pinkish orange or white. **A** Sclerites from colony medulla **B** intermediate form between surface and internal medulla **C** 7-radiates from the surface. Compare this plate with that of Figure 5; these two appear extremely similar, indicating that SBMNH 422978 is likely a color variant (or very bleached) colony of that species represented by SBMNH 422974.

Figures 4 , 5A–C , 6A, B , 7A–C

####### Type locality.

**Holotype** USA, California, Los Angeles County, West end, Santa Catalina Island, 300 meters. **Paratype** USA, California, Los Angeles County, NE × E of Long Point, Santa Catalina Island, 415–486 meters.

####### Type specimens.

**Holotype**SBMNH 422974; **Paratype**SBMNH 422973.

####### Material examined.

~8 lots (see Appendix 1: List of material examined).

####### Diagnosis.

Specimens rarely displayed growth in one plane. Sclerites of medulla with blunt tips, bearing minimal ornamentation, smooth in areas between widely spaced spiny processes; sclerites of colony surface and coenenchymal tissue intermediate between surface and medulla 7-radiates, but never 8-radiates. Thick, compact branches with color variation from pinkish orange to pale pink.

####### Description.

*Colony* (Figure 4, 6) fragments robust, tree-like, with thick, conspicuous branches. Specimen of Figure 4 approximately 18 cm tall, that of Figure 6 roughly 36 cm long (when gently stretched out). Coenenchyme is thick and tough (like cutting through raw carrot). Branches moderately smooth in appearance, although lumpy in many spots, with small calyces evident; appear somewhat moderately spaced, scattered irregularly, on all sides of branches; distal or lateral branch tips each end with round, swollen knob. Color of branch coenenchyme (Figure 4) bright reddish orange; specimen shown in Figure 6 creamy beige with orange polyp apertures (this could be normal color or could have bleached out due to earlier storage solutions); in both specimens, polyps of same orange hue, with tentacles white (more visible in specimen of Figure 4). Cross section samples of both colonies revealed obvious boundary canals, and both colonies have very few, but rather large penetration canals in the medulla. No blunt, stubby, ornate polyp tentacular sclerites (rods) were ever found in any of numerous tissue samples examined; outer surface sclerites are radiate (Figures 5C, 7C), most closely matching a 7-radiate configuration, with ornamentation often jagged and extensive; color of these sclerites a pale pinkish orange; medullary sclerites (Figures 5A, 7A) are spindles, with moderate ornamentation, not as bare as seen in most other species of the genus; these spindles are more or less white, but may often have a very pale yellow color.

####### Etymology.

Nearly all specimens examined, with the exception of two, are from locations within the vicinity of the California Channel Islands, thus a reference to the state of California, where most of the specimens were collected.

####### Common name.

Proposed, “California orange bubblegum” coral.

####### Distribution.

Based on the specimens in the SBMNH collection, ranges from at least Lincoln County, Oregon through southern California waters.

####### Remarks.

Preliminary examinations led to identification of this small group of specimens as *Sibogagorgiacauliflora*Herrera et al., 2010. The two colony morphs shown in their description matched well with that seen in the SBMNH specimens: either a vibrant orange-pink (Figure 4) or a more dull cream color with orange polyp apertures (Figure 6). Unfortunately, the paler-colored specimens at SBMNH had been stored in less than desirable conditions for a lengthy period of time; still, the resemblance was strong. However, the SBMNH specimens were decidedly different in the appearance of their radiate sclerites, taken from the surface cortex. In the species described here, surface radiates were not oval, with 8-radiate origination; this form of sclerite was never seen in examined specimens, despite numerous tissue/sclerite preparations, and is the key distinguishing feature of *S.cauliflora*. Several possibilities emerged: 1) that the SBMNH specimens were an endemic subspecies of *S.cauliflora*, or (because of their strictly southern California location), 2) a very similar, but different species, or 3) the specimens collected were an isolated group of *S.cauliflora* that just happened to be found in a location where, for some environmental reason, the distinctive 8-radiates that normally would form in development and growth, did not. Because of their collection location, and differences in sclerite forms, a new species designation is proposed for what may be a very closely-related, species.

From a taxonomic perspective, in their most recent molecular work Figueroa and Baco (2014) recommended that family Sibogagorgiidae be reinstated. According to WoRMS, the genus *Sibogagorgia* is still retained in the family Paragorgiidae (Cordeiro et al. 2018c).

The museum collection at Moss Landing Marine Labs held a specimen that might be this species, collected in Monterey Bay, 36°26'42"N, 122°01'56"W, ~684 m; coll. G McDonald, 14 August 1974; C0071 [wet]. Coloring of this specimen showed either a slightly bleached condition (storage artifact) or the beige coloring with orange pimples, a slightly brighter condition than that seen in SBMNH 422978.

The SBMNH holotype specimen has, tightly wound around/across its branches, the attachment tendrils from the ends of a swell shark’s mermaid’s purse. The tree-like nature of species not only in this genus, but also in the genus *Paragorgia*, likely provides anchorage and hiding places for a number of organisms, ranging from brittle stars to fish.

###### Family Coralliidae

Taxon classificationAnimaliaAlcyonaceaCoralliidae

Lamouroux, 1812

####### Diagnosis.

Axis an unjointed medullar region composed of completely fused calcareous sclerodermites (solid calcium carbonate) derived from sclerites; Grillo et al. (1993) indicated calcium carbonate axis of family is not derived from the fusion of sclerites, but rather that there are two different origins for the sclerites and the axial skeleton (concentric addition of CaCO_3_). Sclerites present as numerous regular capstans, or as rods, plates, and irregular forms without capstans.

###### Genus Hemicorallium

Taxon classificationAnimaliaAlcyonaceaCoralliidae

Gray, 1867

Madrepora (pars) Linnaeus, 1758: 797.
Corallium [ächte rothe Steincoralle] Müller in Knorr, 1766: Delic. Nat. 1: 7, pl A I, figs 1, 2; 23, pl A VII, fig 1; 24, pl A VIII, figs 2–4; 127 (pars); nec. pp 9–13, 25, 128.
Isis (pars) Linnaeus, 1767: 1288.
Nec Isis Linnaeus, 1758: 799.
Nec Corallium Burman, 1769: [3] (= Isis Linnaeus, 1758).
Corallium Cuvier, 1798[1797]: 673. Lamarck 1801: 378. Dana 1846: 640–641. Gray 1860: 393; 1867: 126. Ridley 1882a: 221–222, 225. Kishinouye 1903: 626; 1904; 28; 1905: 27. Hickson 1905a: 268; 1907b: 13c1, 2. Kükenthal 1919: 743, 828, 902. Aurivillius 1931: 22. Bayer 1950: 61; 1956b: 70, 73; 1964b: 466–467; ? 1993: 17; 1996b: 206, 213. Bayer and Cairns 2003: 222, 224. Tu et al. 2016: 1006.
Pleurocorallium Gray, 1867: 126. Ridley 1882a: 221–222. Johnson 1898: 421; 1899: 59. Kükenthal 1924: 47, 52. Bayer 1956b: 74; 1964b: 467. Bayer and Cairns 2003: 222. Figueroa and Baco 2014b: 83. Tu et al. 2015a: 302; 2015b: 173; 2016: 1022–1023.
Hemicorallium Gray, 1867: 126. Ridley 1882a: 221–222. Johnson 1899: 59. Kükenthal 1924: 47, 52. Bayer 1956b: 74; 1964b: 467. Bayer and Cairns 2003: 222. Ardila et al. 2012: 254. Figueroa and Baco 2014b: 83. Tu et al. 2015a: 302; 2015b: 173; 2016; 1010–1011.

####### Type species.

*Madreporarubra* Linnaeus, 1758 (by subsequent monotypy, the first species being assigned by Lamarck 1801).

####### Diagnosis.

Sclerites of cortex numerous, regular capstans, often modified with six, seven or eight radii; or as double clubs (only some species), crosses and opera glasses; long spindles present in tentacles. Without axial pits bearing beaded rims beneath autozooids. Autozooids prominent, non-retractile (when contracted cannot fully retract into cortex) and ovate-cylindrical, usually distributed on one side of colony.

####### Etymology.

Bayer (1956b) stated that the name *Corallium* “is an old name of dubious origin, going back to the ancient Greeks, classically applied to the red coral of commerce, the ‘true red stony coral’.”

####### Remarks

. An interesting genus; its connection to human enterprise and profit make it so. Collectively, “pink coral, red coral, noble coral, angel skin coral, Sardinia coral, midway coral” (CITES proposal, Convention of the Parties, CoP14 Prop. 21, June 2007) has had a long history, with the primary focus on harvesting of the coral for profit. At least one proposal, and multiple CITES conferences over the years (2007, 2010, 2013), have made this genus a focus of discussion. Numerous articles have been published concerning the impact of harvesting, management issues, etc. (Tsounis et al. 2010, 2013, to name but a couple).

Because all species of coral in this genus tend to form tall, tree-like colonies, they likely increase three-dimensional complexity of the habitats they are found in and consequently, increase biodiversity where they occur. These colonies could easily provide valuable microhabitat for sessile, associated commensal invertebrates (Baco and Shirley 2005, Baco 2007), protecting them from strong currents and predators. With regards to species occurring in the Pacific Ocean, “one of the more notable commensal relationships is the association of polynoid polychaetes with species in (this genus). Each *Corallium* species appears to have its own species of polynoid polychaete, which can reach high densities within individual colonies” (Baco 2007). They would also provide structural relief that fishes and mobile invertebrates could use as feeding, spawning and resting grounds (CITES proposal, Convention of the Parties, June 2007). Thus, they contribute far more to their natural living situation than perhaps had been considered when commercial harvesting for species in this genus was first instituted.

A number of species in the genus *Corallium* have recently been transferred to the genus *Hemicorallium* (encompassing species discussed here; Cordeiro et al. 2019); this is based on the work of Tu et al. (2015a, b, 2016). Species in the genus *Hemicorallium* are represented in the SBMNH collection by a single specimen, SBMNH 471940 (likely *Hemicoralliumducale*; see Appendix 1: List of material examined), but three species have been recorded (collected west of the California Channel Islands), in proximity to the western boundary edge of the Bight (location data for specimens housed at NMNH). The California Academy of Sciences has seven separate lots of specimens in this genus. Generally, all are from the Hawaiian Islands, with the exception of one. That one, identified only to genus, is from California, Davidson Seamount, 128 km SW of Monterey, taken at a depth of 1481 m, 21 May 2002. Further documentation of the presence of *Hemicorallium* in or near the California Bight is needed; in consideration of their rarity and commercial value, their presence would need to be very carefully monitored.

###### Hemicorallium ducale

Taxon classificationAnimaliaAlcyonaceaCoralliidae

(Bayer, 1955)

Corallium ducale Bayer, 1955: 210–211, plate 1. Bayer and Cairns 2003: 224.

####### Material examined.

One lot, 2 specimens + fragments (recent addition) in SBMNH collection, most likely this species (see Appendix 1: List of material examined).

####### Remarks.

Distribution extends in Eastern Pacific from Mexico (Bayer, 1955), northern Baja Mexico (USNM 50111 (type locality) to at least California Channel Islands (USNM 94459). SEM images on file (Bayer’s personal collection): SEM #2284, for USNM 94459 and SEM #s 2483 and 2484 for USNM 50111. Specimen USNM 50111 represented one of the first finds of the genus *Hemicorallium* in North American waters.

The specimen in question does not easily identify to a species; in color it appeared more like that of *Hemicoralliumimperiale*, but the polyps’ appearance and scleritic spindles were more like those seen in *H.ducale*. While all type material for this species (and the other two that follow) at NMNH were examined some years ago, this one specimen, recently received into the SBMNH collection, requires further study.

WoRMS Data Base (Cordeiro et al. 2019) verifies that *H.ducale*, with the other two listed below, are accepted species. They are included, with brief comments, due to their collection locations and proximity to the region of the California Bight. It should be noted that there are no specimens that came into the SBMNH collection from the ‘Velero’ expeditions.

###### Hemicorallium imperiale

Taxon classificationAnimaliaAlcyonaceaCoralliidae

(Bayer, 1955)

Corallium imperiale Bayer, 1955: 209–210, plate 2, c-h. Bayer and Cairns 2003: 224.

####### Material examined.

No material in SBMNH collection (see Appendix 1: List of material examined).

####### Remarks.

Distribution in the Eastern Pacific, northern Baja, California (Bayer 1955), as seen for the **holotype** (USNM 50110) to California Channel Islands, in proximity of western boundary edge of Bight (USNM 85082). No SEM images for this species were found in Bayer’s personal collection at NMNH. Could be called “Imperial red coral.”

###### Hemicorallium regale

Taxon classificationAnimaliaAlcyonaceaCoralliidae

(Bayer, 1956)

Corallium regale Bayer, 1956b: 70, 73–76; 77–78; figs 5c; 7e–g. Bayer and Cairns 2003: 224.

####### Synonyms.

(see Remarks section below.)

####### Material examined.

No material in SBMNH collection (see Appendix 1: List of material examined).

####### Remarks.

Around the Pacific, from Hawaii (holotype, USNM 49520) to offshore seamounts some miles west of California coast (outer edge of Bight western boundary, USNM 94460), certainly at substantial depth (based on specimens housed in NMNH collection). Not enough specimens examined (or collected with attention to specific collection locations) to determine extent of north-south range. Bayer commented (1956b), “of all the Hawaiian precious corals, *C.regale* has the best color and might be of commercial value if it could be fished in quantity.” Thin, calcareous extensions of axis extending outward to thick coenenchyme can support expansion of coenenchyme near sides of branches as recurved flaps, a distinctive feature. These can form tunnels inhabited by polychaete commensals (Baco and Shirley 2005, Baco 2007). Could be given the common name “Regal red coral.”

Several species in the now updated genus *Hemicorallium*, including this one, found within the Hawaiian archipelago (and elsewhere in the western Pacific, including international waters) have been the focus of commercial exploitation (then recognized as species in the genus *Corallium*). Based on reports made public by CITES, regarding “consideration of proposals for amendment of Appendices I and II” (2007, 2010) and Bruckner (2009), there was much discussion, and confusion, as to whether this species (using older genus designation) was valid and/or whether it could be synonymous with *Coralliumlaauense* (misspelled as *C.lauuense*). Grigg and Bayer (1976: 169) and Grigg (2002: 17–19) specifically mentioned *C.regale* and/or *C.laauense* as separate species. CoP14 Prop. 21 (CITES, 2007) listed this species as a potential synonym for *C.laauense*; this based on Baco and Shank (2005: 664). However, Baco and Shank (2005) did not treat this species as a synonym of *C.lauuense* (note the misspelling), but a comment they made regarding the work of Grigg (2002) may have inadvertently lead some to assume that was the case. Cairns (CITES, 2007) did not consider these two taxa to be synonymous and Bruckner (2009: 321) also discussed these taxa as two separate species. Bruckner indicated that although he discussed them as two separate species, “these 2 species may be synonymous.” In the document, Proposal, CoP15 Prop. 21 (CITES 2010), *C.regale* was still shown as a synonym of *C.lauuense* (sic); note the following statement: “*C.lauuense* and *C.regale* are listed as separate species in the US Precious Coral Fishery Management plan, but these species are usually considered synonymous (Parrish 2007)”. Again, *C.laauense* and *C.regale* were treated as synonymous; however, regarding *C.regale*, Baco and Shank (2005) stated: “*C.lauuense* was previously misidentified and referred to as *C.regale* which is not an indication of synonymy. There may still be some unresolved taxonomic problems concerning these two species.” Additionally, “Bayer and Cairns (2003) differs from the SS species list in a number of ways: *C.boshuense*, *C.niveum*, *C.porcellanum*, *C.pusillum*, *C.vanderbilti*, and *C.variabile* are not mentioned; *C.regale* is treated as valid.” (CITES 2010); of these species, *C.boshuense* and *C.variabile* have been moved to the genus *Hemicorallium* (Cordeiro et al. 2019) in the WoRMS Database. The final implication was that *Corallium* sp. was found throughout “the Hawaiian archipelago and into the Emperor Seamount Chain” (Baco 2007), but that the certainty of species identification was still in question.

In studying Bayer’s original 1956 description of *C.laauense* (correctly spelled) and *C.regale*, no determination could be made as to why these two were linked as synonymous. The now recognized species *Hemicoralliumregale* has many double club sclerites, while *H.laauense* has none. As well, the entire colony of *H.regale*, as well as the axis, is pink, while the colony color of *H.laauense* is white or very faintly pink with a white axis. SEM images are on file (Bayer’s personal collection), SEM #2283, USNM 94460.

The work by Tu et al. (2015a, b; 2016) resulted in most species of *Corallium* being placed in the genus *Hemicorallium*. WoRMS (Cordeiro et al. 2019) confirms the placement of the species discussed here: *H.ducale*, *H.imperiale*, *H.regale* (as well as *H.laauense*); all are listed as accepted, separate species.

#### Holaxonia Studer, 1887

With distinct central axis composed of horny material alone or of horny material more or less heavily permeated with calcareous substance, continuous or with alternating horny and calcareous joints. In center of axis is a relatively narrow, largely hollow, tubular space partitioned into series of small chambers, referred to as the cross-chambered central chord. Calcareous material of the peripheral zone of axis is in nonscleritic form (single exception in Keroeididae).

##### Key to Families represented in SBMNH collection (Holaxonia)

**Table:** 

1	Axis horny, with a chambered, hollow, soft central chord	**2**
–	Axis not horny, but is a solid axis, with no soft, central, hollow core	**Suborder Calcaxonia, Part III**
2	Axis purely horny, composed of scleroprotein, without any calcareous deposits	**3**
–	Axis horny, but some calcareous material may be present in some forms; hollow, horny, soft-chambered central chord is wide; there is a peripheral zone of hollow horny spaces containing calcareous material; cortex is thick, with an inner and outer layer, formed by systematic longitudinal canals; polyps retractile into prominent calyces	**Family Plexauridae, Parts II & III**
3	Axis perforated by a wide, cross-chambered central chord; cortex thin; polyps not retractile; sclerites spikey and conspicuous	**Family Acanthogorgiidae, this part**
–	Distinct hollow, horny, soft-chambered central chord that perforates axis is narrow; axial cortex surrounding the core is very dense; polyps fully retractile, into calyces	**Family Gorgoniidae, Part II**

##### List of species of Holaxonia Studer, 1887

Class Anthozoa

Subclass Octocorallia Haeckel, 1866

Order Alcyonacea Lamouroux, 1816

**Suborder Holaxonia Studer, 1887**

Family Acanthogorgiidae Gray, 1859

Acanthogorgiagracillimavar.typica Kükenthal, 1909; (? A.gracillimavar.lata Kükenthal, 1909)

*Acanthogorgia* species A

Muricellacf.complanata Wright & Studer, 1889

##### Descriptions of species of Holaxonia Studer, 1887

###### Family Acanthogorgiidae

Taxon classificationAnimaliaAlcyonaceaAcanthogorgiidae

Gray, 1859

####### Diagnosis.

Axis purely horny (scleroprotein without calcareous deposits), dark-colored, predominantly black; very difficult to cut, with wide, hollow, cross-chambered central core. Coenenchyme very thin, polyps conspicuous, contractile, not retractile, completely covered with both straight and curved fusiform sclerites (forming, in appearance only, cylindrical calyces; no calyces actually present). Sclerites arranged in eight double rows, forming eight en chevron fields; no well-defined operculum; sclerites instead arranged as transverse ring and eight points of converging spindles on tentacle bases; thus, sclerites of polyps continuous with those of tentacular crown, latter being sharp spines arrayed conspicuously around top of polyp, with no intervening sclerite-free neck zone or transverse collaret. Consequently, no clear division between anthocodia and anthostele. Tentacles fold inward over oral disk. Predominant sclerites colorless, in form of prickly or warty spindles; sometimes, presence of three- and four-armed radiates.

###### Genus Acanthogorgia

Taxon classificationAnimaliaAlcyonaceaAcanthogorgiidae

Gray, 1857

Acanthogorgia Gray, 1857a: 128, pl 3, fig 2 [1851]. (pars) Johnson 1861: 297; 1862a: 195. (nec) Verrill 1866a: 152. Pourtales 1867: 113. (nec) Studer 1879: 652 (vide Kükenthal 1919: 911). Verrill 1883: 30. Studer (and Wright) 1887: 54. Wright and Studer 1889: 93 + pl. Hedlund 1890: 8. Studer 1901: 43. Thomson and Henderson 1906a: 50. Kükenthal and Gorzawsky 1908a: 626; 1908b: 52. Kükenthal 1909: 71. Nutting 1910: 12. Kükenthal 1919: 298, 762, 846; 1924: 239. Aurivillius 1931: 53. Stiasny 1943b: 129; 1947: 31. Bayer 1996a: 1–2. Grasshoff 1999: 20; 2000: 40. Fabricius and Alderslade 2001: 184.
Blepharogorgia (pars) Duchassaing & Michelotti, 1864: 15.
Paracanthogorgia Stiasny, 1943b: 130; 1947: 11, 53. Grasshoff 1973: 1; 1992: 89. (Type species: Paracanthogorgiaparatruncata Stiasny, 1943b; species designation by Bayer 1996a: 2).

####### Type species.

*Acanthogorgiahirsuta* Gray, by monotypy (? = *A.aspera* Pourtalès, 1867).

####### Diagnosis.

Colonies generally flattened (flabellate); commonly reticulate, or developed into dense bushy shrubs. Branches appear thin and delicate. Polyps tall, cylindrical, topped with thorny crown of strongly projecting spinous sclerites, embedded at tentacle bases. They lie, collectively, over infolded tentacles, protruding end of sclerites smooth. Polyps on all sides of branches, or roughly biserial; arise vertically at right angle to branch surface, acalycinous, not retractile. Coenenchyme between branches usually thin, axis visible through it. Sclerites in polyps slender spindles slightly bent, arranged en chevron in eight longitudinal double rows. Back of tentacles with only numerous small, flat, bent sclerites; stem coenenchyme with slender, generally bent or sinuous spindles sculptured by prickles or simple tubercles; in deeper layers of coenenchyme (some species), with radiates (tri-radiates and crosses, often with a projecting central spine). Axis dark, but coenenchyme usually colored; sclerites always colorless.

####### Remarks.

Only one collected specimen from the genus appeared in the ‘Velero’ material; it does not match any of the described species it has been compared it to, thus far. It was collected in Mexican waters, beyond the geographic range covered in this work; description of this specimen is in progress.

###### Acanthogorgia gracillima var. typica

Taxon classificationAnimaliaAlcyonaceaAcanthogorgiidae

Kükenthal, 1909

**Figure 8.**
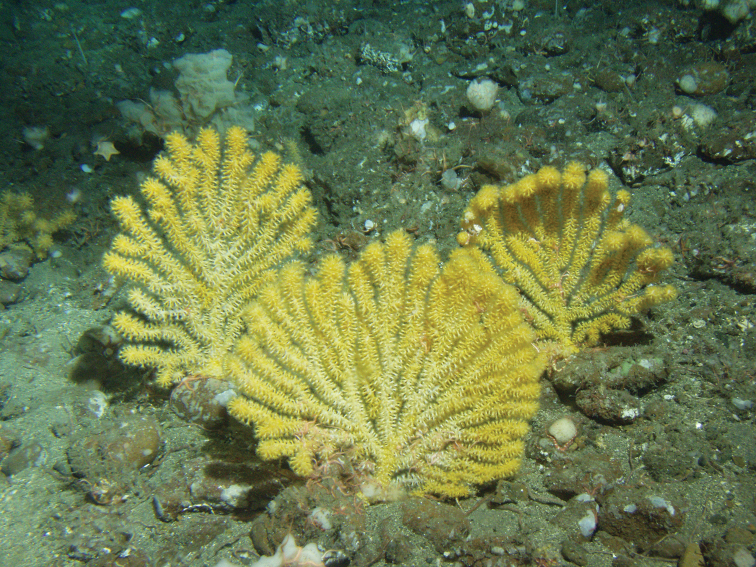
Acanthogorgiagracillimavar.typica. In situ, living colonies, illustrating vibrant yellow color, as seen in Santa Barbara Channel, photograph by D Schroeder; image courtesy of M Love, UCSB.

**Figure 9.**
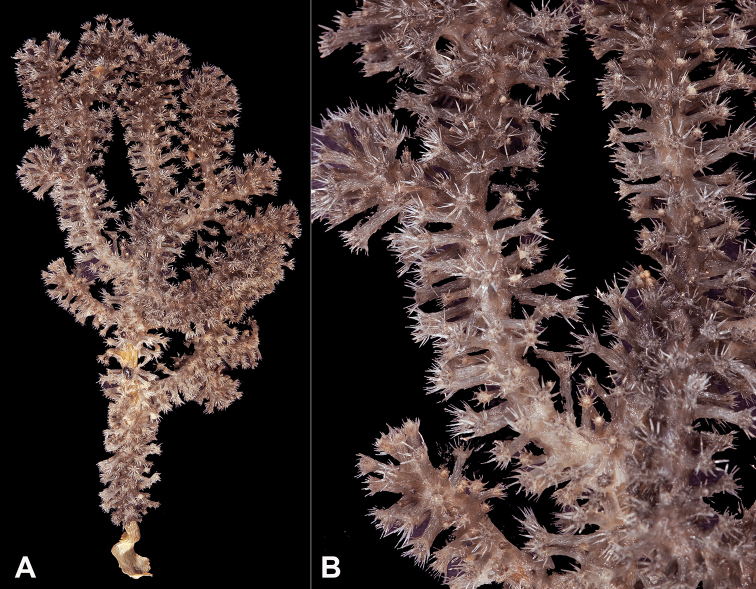
Acanthogorgiagracillimavar.typica, SBMNH 423074. **A** Whole colony preserved in 70% ETOH. Maximum height of colony 15–16 cm, ≤ 10 cm at widest point **B** magnified view of several branches; pronounced, rather spikey appearance of polyps is evident.

**Figure 10.**
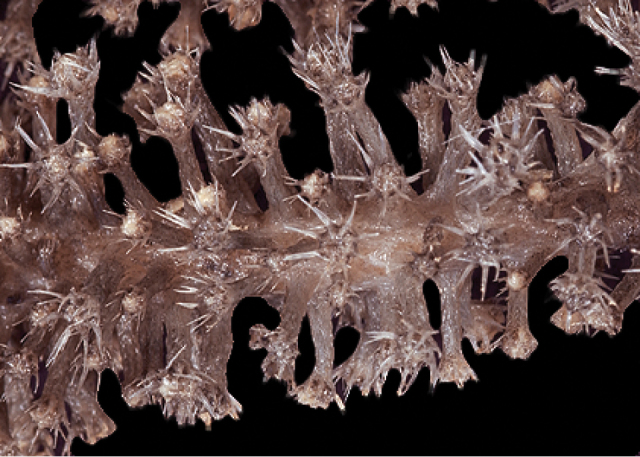
Acanthogorgiagracillimavar.typica, greater magnification of SBMNH 423074. Shows position of polyps on branches. Note prominent crown of sclerites at distal end of each polyp, polyp height averages 5.0–6.0 mm.

**Figure 11.**
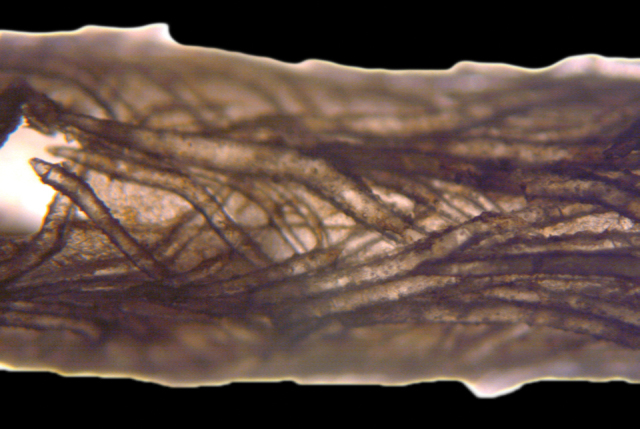
Acanthogorgiagracillimavar.typica (light microscopy image), SBMNH 423074. 4X magnification, demonstrating position of long, boomerang-shaped sclerites, arranged longitudinally on surface of polyp, mid-section to distal end.

**Figure 12.**
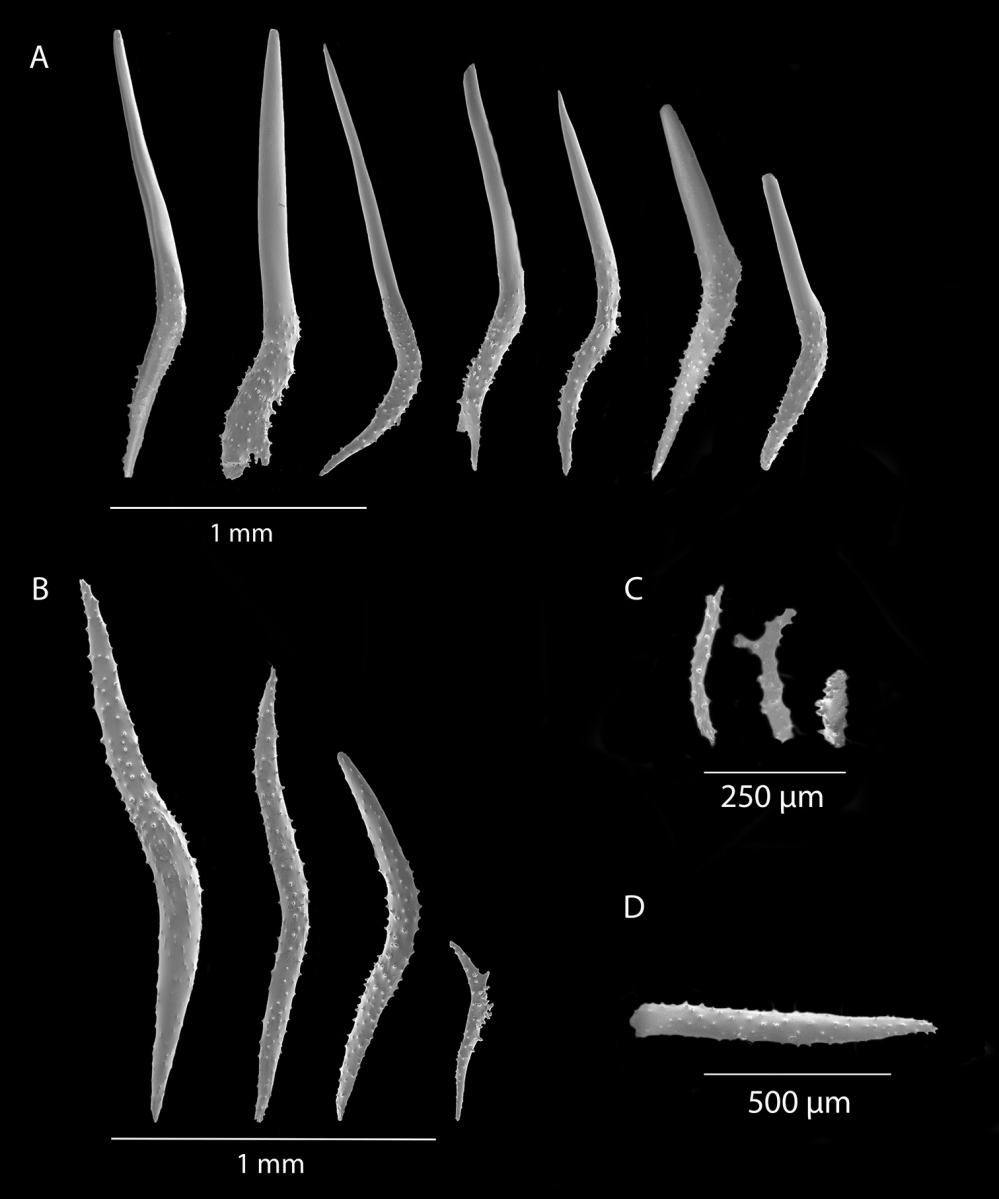
Acanthogorgiagracillimavar.typica, SBMNH 423074, SEM image. These sclerites are colorless. **A** Sclerites from crown **B** sclerites from polyp surface **C** small sclerites from coenenchyme **D** odd coronal or polyp surface sclerite.

Figures 8 , 9A, B , 10 , 11 , 12A–D

Acanthogorgia gracillima var. typica Kükenthal, 1909: 73–76; pl 6, fig 33; (syn.) 1919: 763. Aurivillius 1931: 67–72.
? A.gracillimavar.lata Kükenthal, 1909: 71, 73, 75.

####### Type locality.

(?) Okinawa, Japan; 400 fathoms.

####### Type specimens.

Zoological Museum of the University of Hamburg, Germany (formerly, Naturhistorisches Museum, Hamburg); Catalog Number 3298, with Catalog Number 3297. (Not labeled as type.)

####### Material examined.

1 lot (see Appendix 1: List of material examined).

####### Description.

*Colony* (Figures 8, 9A) richly branched, not entirely in one plane, forming bushy fan or tree no more than 10 cm at widest point. Colony appeared fragile and delicate, but actually a tough, spiky bush/fan, branches reminiscent of those in a bottlebrush (Figure 9B); not greatly flexible. Colony height (dictated by main, central, generally straight stem), base to tip, 15–16 cm; 7.0 cm broad; holdfast remnant present. If any regularity to branching pattern, slightly dichotomous to pinnate, usually in one plane; all lateral branches, of differing lengths, project at nearly right angles, extending/curving quickly upward, even those with more lateral placement. Branch diameter averages 2.0–3.0 mm. Polyps distributed over entire surface (not so much on lower portion of main stem, just above base), nearly in rings around branches, closely placed but not crowded (Figure 10); sometimes two with bases contiguous, generally separated by 1.0 mm, perhaps more; terminal twigs rounded, almost clavate in appearance; numerous polyps at apex, completely covered with straight and curved spindle-shaped sclerites. Polyps not retractile; very conspicuous, decidedly slender, columnar in shape, height between ~4.0–7.0 mm (most average 5.0–6.0 mm tall); diameter generally 1.0 mm for most of polyp length, narrowing slightly, then increasing to ~1.5 mm wide at the crown. Easily recognized by crown of sharp spines encircling top of polyp. Coenenchyme very thin and translucent, with axis color showing through. Color of freshly collected specimen bright lemon yellow (M Love, pers. comm.; Figure 8); on being placed in alcohol quickly turned, generally, light olive-green towards base, becoming slightly darker grey-green at uppermost branch tips. (While species in family are described as having a predominantly black, purely horny axis, color of axis showing through extremely thin coenenchyme appeared to account for overall olive-green color. Having now sat in 70% ETOH for some time, the colony has turned more yellowish brown.) Sclerites (Figures 11, 12A, B) mainly spindle-shaped; straight or curved, showing arrangement of eight double rows, forming longitudinally-placed chevrons (obliquely angled double-rows) characteristic of genus. A very few oddly branched; some, more a tripod shape. Sclerites appear mostly tuberculate, with distinct boomerang bend (Figure 12A), easily removed from surface of colony. The longest sclerites, with distinct bend roughly a third of the way along their length, range from 1.0–2.0 mm in length (average 1.6 mm L × 0.17 mm W); one third of surface of sclerite bears tubercles, while other two-thirds is generally smooth; this smooth section, thin, rounded, somewhat beveled, is the distal, prominent spike that projects from the thorny basal portion embedded in the mesoglea of the body wall, in nearly longitudinal direction; lower, embedded portion, ~0.5 mm long, appears to cross over into the neighboring angled rows, these basal portions not much different one from another. These sclerites form the crown of thorns seen around top of polyp. Two very long spines project upwards to form the points of the crown at distal end of each of the eight double-rows. Smooth portion of these sclerites sit with approximately 1.0 mm of their length free of polyp. Numerous, slightly smaller, flatter sclerites have bend more centrally located, their entire surface covered with tubercles (average 1.0 mm L × ~0.08 mm W). These primarily cover outside surface of polyp (Figure 11), illustrating chevron pattern (eight longitudinal double rows) in placement. Sclerites of coenenchyme similar to those seen on polyps’ surface (~0.8 mm long), perhaps slightly thicker in width and slightly more fully covered with tubercles; tentacular sclerites smaller still, bent, flattened (up to ~0.18 mm long), completely covered with tubercles; more prominent, dense, on dorsal side of tentacles. If present, a few smaller (0.1 mm long) radiate or cross-shaped sclerites may be seen. In the coenenchyme covering the base, exclusively, are 0.25 mm long, bent, strongly spined spindles. All sclerites completely colorless, reminiscent of thin, bent shards of glass.

####### Etymology.

For designation *gracillima*, the Latin *gracili*- means slender; this may reference the conspicuous, very slender polyps and very slender points in the crown of this species. Kükenthal (1909) gave no explanation for the derivation of the species name.

####### Common name.

None specifically designated; the Slender glass-shard gorgonian would be appropriate. Worth considering is whether or not this is the species that, predominantly, MBARI and NOAA researchers are seeing and calling the “Gold gorgonian.“

####### Distribution.

For genus in general, “species. . . inhabit moderate to considerable depths; various species of *Acanthogorgia* occur in all seas, some thriving in very cold waters” (Bayer, penciled personal annotations in Kükenthal 1924). Examinations of the literature revealed numerous species in the Indo-Pacific region. Recent MBARI on-line postings indicated this genus is found in northern California waters, often at great depth. Milton Love (description based on specimen he collected) indicated (pers. comm.) that the color of this gorgonian is very vibrant, thus easily seen; it is quite abundant in certain areas, such as the Footprint, a feature outside the Anacapa/Santa Cruz Island Passage. Thus, for this species, range seems to extend around the Pacific Ocean from Japan (Kükenthal’s specimen) to eastern Pacific waters of California; further study will be required to determine whether, and how far, it extends north and south of California.

####### Biology.

On this particular specimen, there were at least two scale worms wrapped around the base of polyps in two separate areas within the colony.

####### Remarks.

To handle the specimen during examination, although colony fairly hardy, gloves were necessary. Generally, all the long sclerites were very sharp, comparable to small shards of thin glass; those that got into the skin under the fingernail were rather painful. Given the delicate, and somewhat brittle/fragile nature of the longer boomerang-type sclerites, these sclerites are easily broken. It was often difficult to ascertain whether radiates and crosses really existed as such, or whether they were bits that had broken off from the longer spindle forms; this could account for some of the odder-shaped sclerites that previous description (Kükenthal 1909) made mention of. Microscopic examinations were always done without a cover glass, when possible, so that sclerites were not crushed.

This description (and original description for the species) was based on the specimen used by Kükenthal (1909). In initial comparisons of known, and described species, using characteristics of the colony (lying more or less in one plane, with polyps generally more than 4.0 mm in length), the key that Kükenthal (1924) provided in his overview of the genus was relied upon, but it took some time to rule possibilities out. Bayer (1996b) stated that all the sclerites are so similar in form within all members of the genus, that distinguishing species is difficult; this was confirmed. As well, there are very few recent descriptions of any *Acanthogorgia* species that are truly adequate to use for comparative purposes. Few specimens had been collected from California waters; thus, not a lot of material to compare against. In examinations of collections at other institutions, only two/three specimens were found at NMNH that approximated the appearance of this colony (USNM 1071429, dry and USNM 1072361, wet, in two lots). The dry specimen is from Cross Seamount, Hawaii, USA and the wet lots are from a seamount east of Necker Island, Hawaii, USA. Both are identified to genus, but not to species; in color, branch pattern, pattern of sclerite positioning on polyps and sclerite morphology, specimen USNM1071429 appeared most similar. If NMNH specimens can be accurately identified to species, they could confirm (or negate) the notion of trans-Pacific distribution. In any event, this discussion/description represents one of the first published reports for this particular species in southern to central California waters; overall the genus needs further work, not only on those specimens being frequently noted in underwater explorations (cruises and surveys being done by MBARI and NOAA), but also on those that have already been collected, perhaps described or need description, currently housed in other museum collections. With greater access to locations of depth, and the use of ROV, AUV, digital imaging and improved ability to collect specimens at depth, many currently described species can be confirmed and many new species are likely to come to light. As well, with careful attention to locations where specimens are found, it should be possible to develop a far more accurate picture of the distribution of these deep-water forms.

While the original specimen does indeed still exist, no opportunity to travel to Hamburg, Germany occurred and no request that the specimen be sent was made. The species is an accepted species in the WoRMS Data Base (Cordeiro et al. 2018e).

[***Acanthogorgia* (= ? *Acalycigorgia*) Grey, 1857)**]; not accepted, WoRMS (Cordeiro et al. 2018e).

###### Genus Acalycigorgia

Taxon classificationAnimaliaAlcyonaceaAcanthogorgiidae

(= ? Acanthogorgia) Kükenthal, 1908; not accepted, WoRMS (Cordeiro et al. 2018e)

Acalycigorgia Kükenthal, 1908b: 38; 1919: 298; 1924: 237–239. Kükenthal and Gorzawsky 1908a: 629; 1908b: 38. Kükenthal 1919: 764, 846. Aurivillius 1931: 40. Bayer 1956a: F203; 1981c: 920.
? = Acanthogorgia Gray, 1857a: 128, pl 3, fig 2 [1851]. (pars) Hedlund 1890: 3, 6. (pars) Thomson and Russell 1910: 145. Fabricius and Alderslade 2001: 184.
? Paramuricea Moroff, 1902: 407.

####### Type species.

*A.grandiflora* Kükenthal & Gorzawsky, 1908a; subsequent designation by Kükenthal and Gorzawsky (1908b).

####### Diagnosis.

Polyps not functionally differentiated into anthocodia and anthostele; contractile but not retractile within common coenenchyme; tentacles fold over oral disk in contraction. Polyps similar to those of *Acanthogorgia* (without crown of strongly projecting spines, however), but polyps can be short and verruciform to prominent, tall and cylindrical, not clavate. Sclerites of polyp walls large spindles, very conspicuous; commonly arranged more or less distinctly en chevron in eight long, longitudinal double rows, but distal ones project little or not at all. Distal ends of sclerites around tentacle bases not specifically differentiated as spines, though the tips may project somewhat around polyps’ apex. Polyps are without suture separating tentacular/anthocodial from subtentacular sclerites. Sclerites of polyp body gradually merge with those of tentacle bases, which are not abruptly smaller; coenenchymal sclerites with tubercles of inner and outer sides similarly developed; inner layer of coenenchyme with more or less abundant radiates.

###### Acanthogorgia

Taxon classificationAnimaliaAlcyonaceaAcanthogorgiidae

species A

**Figure 13.**
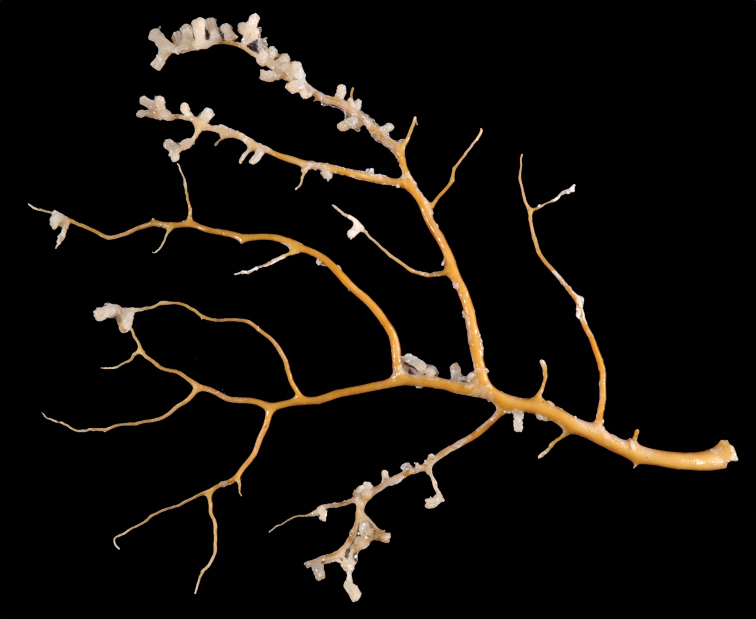
*Acanthogorgia* species A, SBMNH 423075. Of tissue present, polyps readily visible, colony measuring ~ 9.0 cm in height.

**Figure 14.**
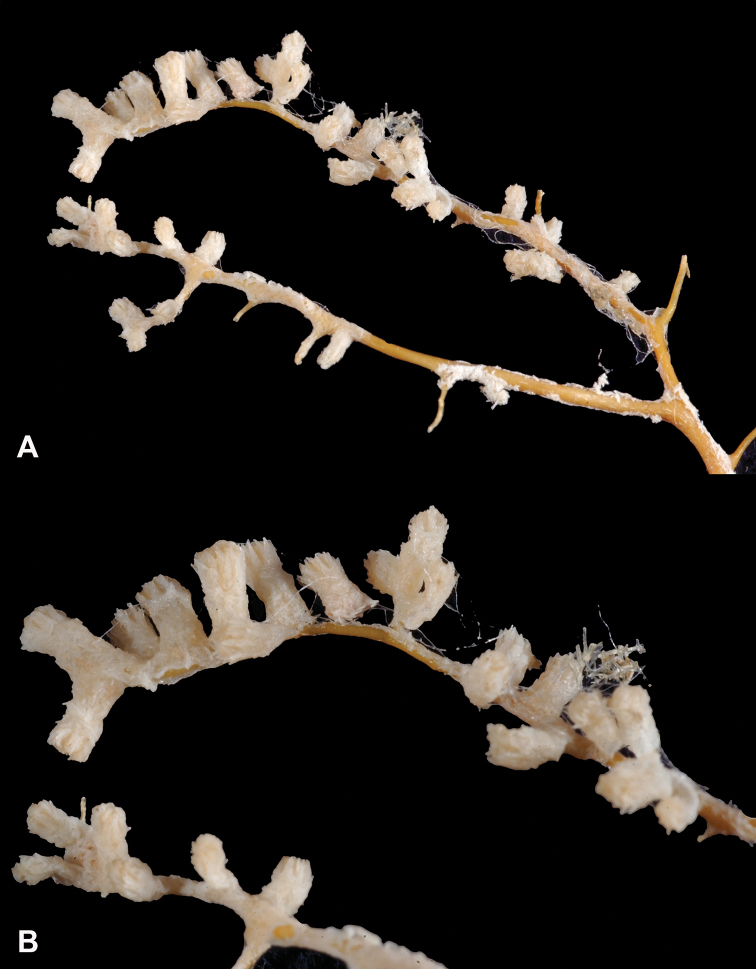
*Acanthogorgia* species A, SBMNH 423075. **A** Closer view of polyps present at tips of several branches; polyp height 3.0 mm tall, 1.0 mm broad **B** further magnification of polyps. Barely visible at distal end of several polyps, the weakly developed crown of sclerites can be seen.

**Figure 15.**
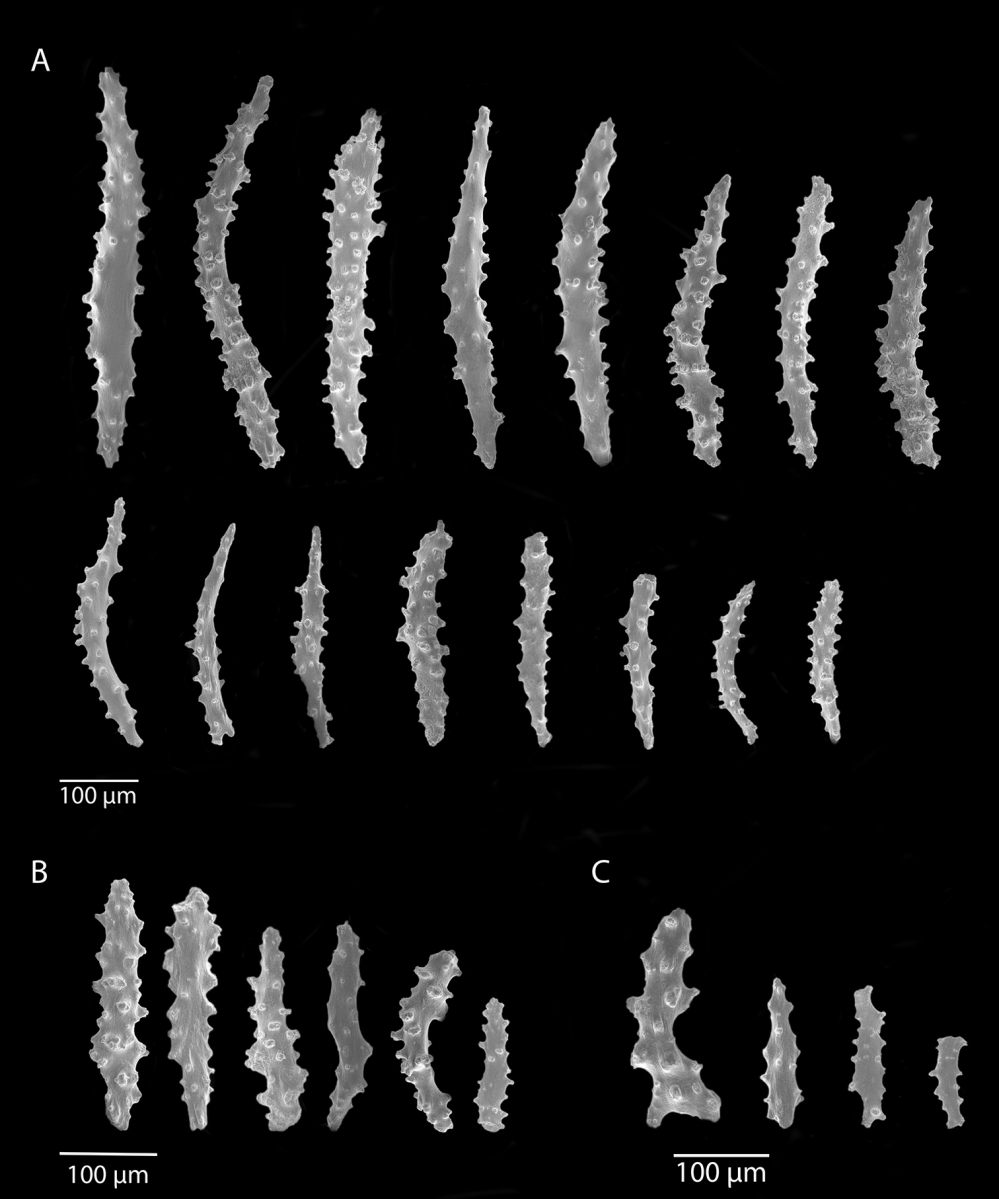
*Acanthogorgia* species A, SBMNH 423075, SEM image. Sclerites from these colonies are white, rather than clear. **A** Coronal sclerites **B, C** smaller coenenchymal sclerites.

Figures 13 , 14A, B , 15A–C

####### Type locality.

Cannot be indicated at this time.

####### Type specimens.

Cannot be indicated at this time.

####### Material examined.

1 lot (see Appendix 1: List of material examined).

####### Description.

*Colonies* (two) generally in one plane; one measures 7.0 cm × 5.5 cm (length to width); second (Figure 13) measures 9.0 cm × 4.5 cm, at widest, halfway up colony. Thin, delicate-looking branches (round to slightly square in shape); branching more or less dichotomous; closely monopodial. No flattening at branch origins. Base 2.0 mm wide, main branch 1.0 mm wide; branchlets vary between 0.5–0.75 mm wide and tips of branchlets very thin, thread-like; all branches quite stiff. Coenenchyme very thin (very little still present in these specimens); axis predominantly exposed, yellow-gold to rusty-brown. Of the few polyps present on a few branches, most located near branch tips (Figure 14A); coenenchyme and polyps creamy-white. Polyps primarily sit lateral to branch, at distance of ~1.0 mm or less from each other; closer to branch tip sitting literally side by side; some few branches indicate that polyps can be found on all sides. There are marked, longitudinal grooves/ridges at distal ends of polyps; there is barely apparent a very short little spiny crown at their very tip (Figure 14B). The ridges, eight in number, are each formed by a parallel collection of two or three bent spindle-type sclerites. Polyp surface densely covered with sclerites; no calyx apparent. Polyp height 3.0 mm, 2.0 mm from base to area of longitudinal grooves with another 1.0 mm of height when area of grooves/ridges included. All approximately 1.0 mm wide, distal end slightly wider, somewhat obvious, ~1.5 mm wide. No expanded tentacles readily visible (contracted over mouth); all heavily covered or encased by sclerites. Sclerites (Figures 15, A particularly) predominantly bent spindles; all tuberculated across entire surface, averaging 0.5 mm long by 0.08 mm wide. The largest (~0.7 mm × ~0.1 mm), decidedly bent spindles; these form the eight ridges mentioned above; others appear to lie in longitudinal direction up to and beyond upper edge of polyp, barely showing as short points of a crown. Bent spindles, somewhat smaller, almost tend to the formation of the en chevron, double-row pattern at the proximal end of ridges and down on to lower end of polyp. Also, less bent ones, seemingly very narrow spindles (0.6 mm × 0.05 mm); few appear slightly club-shaped (average 0.4 mm × 0.06 mm), primarily from lower polyp wall and coenenchyme. Sclerites with boomerang shape scarce or not present. From initial light microscopy examination, apparent that many of these spindles can be broken; many odd-shaped bits seen in arrays, with some of the spindles having oddly truncated ends, where some aspect of the sclerites likely had broken off. All forms quite densely arrayed on specimen’s surface, giving polyp and branch coenenchyme a distinct white to glassy appearance; all sclerites colorless. Inner coenenchyme radiates not found.

####### Remarks.

It is possible that these specimens, and several others in the collections either at CAS or NMNH, Smithsonian (not yet identified to species), could be first records of appearance for this species in, or near boundaries of, the California Bight. Description here is based on two specimens, which in overall colony appearance superficially look very similar to Nutting’s photographs (1912: pl 11, fig 1, 1a), shown for *Muriceidescylindrica*, which Nutting established as a new species. However, the sclerites illustrated in Nutting (1912: pl 20, fig 3), while of comparable size to that indicated for the species described here, do not match. In considering overall appearance of polyps on the branches, and the manner in which sclerites covered the surface of the polyps from distal to proximal ends, a generic best fit occurred working from an illustration that was given in Bayer (1956a) for *Acalycigorgia*. However, Fabricius and Alderslade (2001) have stated that “(m)any species of *Acanthogorgia*, in which the polyp sclerites are so short that there is little or no projecting crown, erroneously appear in the literature under the name *Acalycigorgia* Kükenthal, 1919. *Acalycigorgia* is actually a synonym of *Acanthogorgia* and most species referred to the former should be called *Acanthogorgia*.” This is supported in the WoRMS Data Base (Cordeiro et al. 2018e) for taxon information regarding status of the genus *Acalycigorgia*. It now remains to be seen if the species described here is indeed one of the 61 species listed as accepted by the Data Base.

###### Genus Muricella

Taxon classificationAnimaliaAlcyonaceaAcanthogorgiidae

Verrill, 1869

Lissogorgia (pars) Verrill, 1865: 187.
Muricea (pars) Verrill, 1868c: 411–416.
Muricella Verrill, 1869a: 450 (= ? Anthogorgia Fabricius & Alderslade, 2001). Studer 1879: 650. Ridley 1882b: 128; [= ? MuriceaRidley 1884: 335, 579]. Studer 1887: 58. Wright and Studer 1889: 123 + plate. Germanos 1896: 181. Brundin 1896: 17. Whitelegge 1897: 315. Hiles 1899: 49. Hargitt and Rogers 1900–1902: 282. Hickson 1905b: 815. Thomson and Henderson 1905a: 301; 1905b: 178; 1906a: 78. Nutting 1908: 586; 1909: 717. Thomson and Simpson 1909: 241. [= ? VersluysiaNutting 1910: 29, 35]. Thomson and Russell 1910: 158. Nutting 1912: 78. Kükenthal 1913b: 28. Schimbke 1915: 26. Kükenthal 1919: 75, 838, 909. Molander 1921: 11. Aurivillius 1931: 123. Bayer 1956a: F206. Grasshoff 1999: 33. Fabricius and Alderslade 2001: 188.
? Anthogorgia Verrill, 1868 (=?Acalycigorgia Kükenthal, 1908b). Grasshoff 1999: 33. Fabricius and Alderslade 2001: 186.
? Acalycigorgia Kükenthal, 1908b (= ? Acanthogorgia or Astrogorgia [see Fabricius and Alderslade 2001] as proposed by Cairns and Bayer, unpublished synonymy).
? Astrogorgia Verrill, 1868c: 413. Fabricius and Alderslade 2001: 210.
? Acanthogorgia Gray, 1857a: 128 [1851]. Fabricius and Alderslade 2001: 184.

####### Type species.

*Lissogorgiaflexuosa* Verrill, 1865; [subsequent diagnosis by Nutting 1910].

####### Diagnosis.

Colonies fan-shaped, branching in one plane, some anastomosing. Larger branches with axis often tending to be flattened at right angles to plane of fan; in older colonies, smaller branches can bend and grow perpendicular from the fan. Polyps prominent, low, wart-like, non-retractile; coenenchyme thick between polyps. Sclerites of polyp tentacles small rods; below anthocodiae, sclerites large and bow-shaped, in angled double rows (chevrons), forming eight marginal points, forming strong collaret; tentacular operculum distinct. Sclerites of coenenchyme in two distinct layers, mostly spindles (coarse or densely warted), small capstans, with some clubs or discoidal forms.

####### Remarks.

Kükenthal (1924) articulated the characteristics of this genus, which include: condition of the branch ends, position, orientation, height, and form of polyps, presence of an operculum, and arrangement of sclerites in the coenencyme. Fabricius and Alderslade (2001) discussed aspects of comparison/contrast between this genus and others; it was stated, “not much separates this genus from *Anthogorgia*, and a study of a large suite of specimens could see the two synonymized.” Additionally, a characteristic feature pertinent to the genus diagnosis came from the Octocoral Taxonomy Laboratory Manual (2007): There are no calyces in the genus *Muricella*; “almost 90% of the species attributed to this genus do not belong there. A major revision is needed.” In reviewing older literature there were references to calyces in this genus; Bayer (1956a) comments on the “truncated cones” or “rounded, low verrucae.” In descriptions given by Verrill (1869a), reference is made to calyces, as well. The concept of how to define a calyx may need revisiting, notably with regards to this genus.

###### Muricella complanata

Taxon classificationAnimaliaAlcyonaceaAcanthogorgiidae

Wright & Studer, 1889

Muricella complanata Wright & Studer, 1889: 125–126. Thomson and Henderson 1905a: 303. Nutting 1909: 717–718. Thomson and Simpson 1909: 250. Nutting 1910: 31. Thomson and Russell 1910: 158. Kükenthal 1924: 172.

####### Material examined.

No material identified as this species in collection at SBMNH (see Appendix 1: List of material examined).

####### Remarks.

Included here briefly, as there was a California collection location for a specimen that was identified as this species.

Based on Kükenthal (1924), the species ranges from coastal to abyssal depths; likely can be found in a wide and diverse number of locations around the world. The type was collected off Japan, but present location of the type specimen could not be determined. The specimen upon which Nutting’s 1909 description was based, taken by the Fisheries Steamer ‘Albatross’, Station 4461, Point Loma Light-house, S 3, E 9.3 miles, 285 fathoms, was said to have differed slightly from the type, particularly in having a well-marked collaret and in the arrangement of the calycular sclerites. Kükenthal (1924) indicated that the California location (at an approximate depth of 250 m) is questionable. There is no certainty that any specimens found in the California Bight will be specimens of *Muricellacomplanata*, or whether the species actually exists. Harden (unpublished dissertation, 1979) suggested that *Muricellacomplanata* was synonymous with *Swiftiatorreyi*. Both were stated to display anastomosing (*S.torreyi* is always anastomosed; was unable to determine if that is always case with *Muricellacomplanata*, as thus far no confirmed specimen of this species has been located). As well, I would disagree with a comment found in an unpublished Bayer annotation: “*Muricellacomplanata* = a *Swiftia*?” in that *S.torreyi* is a plexaurid, while *Muricella* is a recognized genus within the family Acanthogorgiidae (Bayer 1981c, Fabricius and Alderslade 2001, Cordeiro et al. 2018f) with no mention of any species of *Muricella* being synonymized with any *Swiftia* species.

## Conclusions

It is clear from this first part of the systematic review of alcyonacean gorgonian species in the SBMNH collection that the Scleraxonia and the family Acanthogorgiidae (Holaxonia) are found within the California Bight but are not well-represented within the collection; as well, very few of the species examined for this first part came to the museum via the Allan Hancock Foundation ‘Velero’ Expeditions. This is not surprising; many of the species in question would be/are fairly deep water forms, and the technology for achieving the required depths was not available at the time of those expeditions. As well, much of the ‘Velero’ material came from locations located closer to shore, geographic areas that the ‘Velero’ expeditions primarily focused on and worked in; many species of scleraxonian, as well as those of the holaxonian Acanthogorgiidae, generally exist in deeper water further off of mainland California, particularly in canyons and basins associated with the California Channel Islands. Despite visiting several institutions where these groups were better represented, there was simply not enough material in the SBMNH collection to make good comparisons. As well, there were not enough specimens in the SBMNH collection to adequately display the variation within and across species within the discussed families. Based on survey work done by NOAA in the recent past as part of their Deep Sea Coral Research and Technology Program, West Coast Research Initiative (supported by the Magnuson-Stevens Fisheries Conservation and Management Act of 2006), with field research programs undertaken off California, Oregon and Washington in 2010–2012, and during the years 2016 and 2017, more and more material from the groups covered in this first part is being photographed, mapped and collected. From these two major west coast research events, it is clear that there are many more species in the groups discussed here, and thus a far greater degree of octocoral biodiversity appearing off the California coast, at greater depths, than is evidenced by the SBMNH collection. Additionally, the West Coast Deep-Sea Coral Initiative for fiscal years 2018–2021, which is just beginning to establish priority collecting sites in preparation for this segment of exploratory research, will reveal much more, as the goal is to explore even greater depths at strategic sites that have not yet been extensively studied. As more of this exploratory and survey work is undertaken within, or near, the region of the California Bight, primarily by NOAA and its collaborators, there will be a need for taxonomic work, and through that work (some of which will be conducted by myself, in collaboration with staff at several NOAA field offices), the museum may be able to increase its specimen holdings to better illustrate these taxonomic groups as they relate to gorgonian diversity in the California Bight.

## Supplementary Material

XML Treatment for Anthothelidae

XML Treatment for Anthothela

XML Treatment for Anthothela pacifica

XML Treatment for Paragorgiidae

XML Treatment for Paragorgia

XML Treatment for Paragorgia arborea var. pacifica

XML Treatment for Paragorgia regalis

XML Treatment for Paragorgia stephencairnsi

XML Treatment for Paragorgia yutlinux

XML Treatment for Sibogagorgia

XML Treatment for Sibogagorgia californica

XML Treatment for Coralliidae

XML Treatment for Hemicorallium

XML Treatment for Hemicorallium ducale

XML Treatment for Hemicorallium imperiale

XML Treatment for Hemicorallium regale

XML Treatment for Acanthogorgiidae

XML Treatment for Acanthogorgia

XML Treatment for Acanthogorgia gracillima var. typica

XML Treatment for Acalycigorgia

XML Treatment for Acanthogorgia

XML Treatment for Muricella

XML Treatment for Muricella complanata

## Appendix 1

**List of material examined – Part I**

(Material examined = whole colony study plus multiple sclerite preparations; all with light microscopy, plus selected colonies under SEM, shown in figures associated with text)

***Anthothelaargentea* Studer, 1894**

**Material examined**. No specimens in SBMNH collection.

**Other material, not examined.** – ±1 colony; USA, off California coast, west of Channel Islands, Fieberling Guyot, 32°27'36"N, 127°47'36"W, 490 m; coll. R/V ‘Alvin’, 10 October 1990; USNM 94428 [wet].

***Anthothelapacifica* (Kükenthal, 1913)**

**Material examined.** 1 lot **USA, California** – 1 colony; San Diego County, edge of ridge running through Rodriquez Seamount, slightly northwest of West Cortes Basin, due west of southern tip, San Clemente Island, on sponge, 32°37'43"N, 120°05'04"W, 950–1150 m; coll. skipper of vessel ‘Calafia’ (California Department of Fish and Game – Long Beach, California), 9 August 1978; SBMNH 265939 [wet].

**Other material examined.** – ±1 colony; USA, California, San Diego County, San Diego, Point Loma, ~32°40'38"N, 117°15'08"W, 201–262 m; coll. R/V ‘Albatross’, 4 March 1904; USNM 49519 [wet]. (Additional label in jar indicated “*Anthothelaargentea*.”)

**Paragorgiaarboreavar.pacifica (Verrill, 1922)**

**Material examined.** ~2 lots **USA, California** – 1 branch fragment; Orange County, San Clemente Island, between Pete Bay, 3–10 miles E X (N+S) of island, ~32°55'48"N, 118°24'19"W, 1000 m; coll. unknown, 19 July 1978; SBMNH 422977 [wet]. –2 branch fragments; California, Monterey County, Monterey Bay, 5.8 miles, 314° T from Point Pinos Light to Mid Point, 36°43'00"N, 122°01'45"W, 436–809 m; coll. R/V ‘Velero IV’, stations 7462-61 or 7463-61, 10 October 1961; SBMNH 422976 [wet].

**Other material examined.** – 1 colony/fragment; USA, California, Ventura County, off San Nicolas Island, (from fishermen’s net); coll. unknown, March 1985; donated by R Williams, of “The Sea;” CBA #85.14.2 [dry]. -- USA, California, Monterey County, Davidson Seamount, 1,313 m; coll. unknown, 2003; USNM 1014919 [wet].

***Paragorgiaregalis* Nutting, 1912**

**Material examined**. No apparent material in SBMNH collection.

**Other material examined.** – 1 colony fragment; USA, California, Santa Barbara County, west of San Miguel Passage on Rodriquez Seamount, 33°57'12"N, 121°08'41"W, 1,840 m; coll. unknown, 14 October 2003; USNM 1027063 [wet].

***Paragorgiastephencairnsi* Sánchez, 2005**

**Material examined**. No material in SBMNH collection.

**Other material examined.** – 1 colony; USA, California, west of California Channel Islands, Fieberling Guyot, 36°26'00"N, 127°47'36"W, 490 m; coll. DS/V ‘Alvin’, 16 October 1990; USNM 94437 (**Paratype**) [wet].

***Paragorgiayutlinux* Sánchez, 2005**

**Material examined**. No material in SBMNH collection.

**Other material examined.** – 1 colony; USA, California, west of the California Channel Islands, Fieberling Guyot, from summit of seamount, 32°26'00"N, 127°47'42"W, 487 m; coll. DS/V ‘Alvin’, 21 October 1990; USNM 90345 [wet].

***Sibogagorgiacalifornica* sp. nov.**

**Material examined.** ~6 lots **USA, California** – 1 branch fragment; Los Angeles County, Santa Catalina Island, 7 miles WSW of Church Rock, substrate loose rock, 33°14'40"N, 118°11'10"W, 276–282 m; coll. R/V ‘Velero III’, station 1323-41, 18 May 1941; SBMNH 422971 [wet]. –1 branch fragment; Los Angeles County, 5.5 SE of Santa Catalina Island, on boulders and gravel, 33°15'30"N, 118°12'50"W, 264–273 m; coll. R/V ‘Velero III’, station 1172-40, 20 August 1940; SBMNH 422972 [wet]. –1 branch fragment; Los Angeles County, Santa Catalina Island, 6.25 miles NE or E.NE X E of Long Point, with rocks, sponges, cyclostomes, 33°25'20"N, 118°14'40"W, 415–486 m; coll. R/V ‘Velero III’, station 1400-41, 8 September 1941; SBMNH 422973 [wet]. –1–2 branch fragments; Los Angeles County, Santa Catalina Island, 5.7 miles, 123° T to West End, 33°30'55"N, 118°43'15"W, 300 m; coll. R/V ‘Velero IV’, station 24471-76, 8 March 1976; SBMNH 422974 [wet]. –multiple branch fragments; Los Angeles County, Santa Barbara Island, 6.7 miles, 330° T from N Light, dredge, tangles (2 large boulders with much small rock, rock bottom), 33°33'27"N, 119°04'00"W, 255 m; coll. R/V ‘Velero IV’, station 2062-51, 18 October 1951; SBMNH 422975 [wet]. **USA, Oregon** – multiple branches; Lincoln County, 55.98 miles W of coast, 44°33'30"N, 125°12'42"W, 1600 m; coll. “Oregon State University—R/V ‘Yaquina’, Cruise Y6810C,” 15 October 1968; SBMNH 422978 [wet]. –1 large colony; off coast, 46°06'22"N, 124°55'03"W, 1123 m; coll. Astoria RB-01-05, G Hendler, RV ‘Ronald H. Brown’ (NOAA) and S/V ‘Ropos’, Dive R602-Bio-0008, 3 July 2001; LACoMNH Marine Biodiversity Center #374 [wet].

***Hemicoralliumducale* (Bayer, 1955)**

**Material examined.** One lot (possibly this species). **USA, California** – 2 colonies + fragments; USA, California, Los Angeles County, Catalina Basin, N of San Clemente Island, 33°04'49"N, 118°29'52"W, 1,091 m; coll. P Gregory, California Fish and Game, Long Beach, 15 November 1977; SBMNH 471940 [wet].

**Other material examined.** – 1 colony; USA, California, West of Channel Islands, Fieberling Guyot, “Sea Pen Rim,” 32°27'36"N, 127°49'30"W, 640 m; coll. DS/V ‘Alvin’, 14 October 1990; USNM 94459 [wet].

***Hemicoralliumimperiale* (Bayer, 1955)**

**Material examined.** No material in SBMNH collection.

**Other material examined.** – multiple fragments; USA, California, Fieberling Guyot Seamount, 32°23'00"N, 127°47'00"W, ~600 m; coll. unknown, August 1989; USNM 85082 [wet].

***Hemicoralliumregale* (Bayer, 1956)**

**Material examined**. No material in SBMNH collection.

**Other material examined.** –1 colony; USA, California, Davidson Seamount; 1,482 m; coll. unknown, 2003; USNM 1014918 [wet]. –fragment; USA, California, west of Channel Islands, Fieberling Guyot, 32°27'36"N, 127°49'30"W, 640 m; coll. DS/V ‘Alvin’, 7 December 1990; USNM 94460 [wet].

**Acanthogorgiagracillimavar.typica Kükenthal, 1909**

**(? A.gracillimavar.lata Kükenthal, 1909)**

**Material examined.** 1 lot **USA, California** – 1 colony; California, Ventura County, Channel Islands, 33°57'35"N, 119°28'34"W, 160 m; coll. M Love from submersible ‘Delta’, 8 October, 2005; with his number: A6662; SBMNH 423074 [wet].

**Other material examined.** – 1 fragment (multiple colonies); USA, northern Pacific Ocean, Hawaii, Cross Seamount, 18°43'55"N, 158°15'41"W, 388.56 m; coll. Smith, 10 October 2004; USNM 1071429 [dry]. –1 colony; USA, northern Pacific Ocean, Hawaii, Cross Seamount, 18°41'17"N, 158°17'58"W, 477 m; coll. RB Moffitt and EH Chave, 6 February 2003; USNM 1014741 [dry]. –multiple colonies; USA, northern Pacific Ocean, Hawaii, Necker Island, Seamount east of island, 23°13'55"N, 163°31'07"W, 1720 m; coll. A Baco-Taylor, 1 November 2003; USNM 1072361 [wet]. –(?) multiple colonies; USA, northern Pacific Ocean, Hawaii, Pioneer Bank, 25°34'27"N, 173°30'22"W, 1,743.7 m; coll. A Baco-Taylor, 9 October 2003; USNM 1072344 [wet].

***Acanthogorgia* species A (= ? *Acalycigorgia*)**

**Material examined.** 1 lot **USA, California** – 2 colonies; Monterey County, Monterey Bay, from Point Piños Light to Mid Point, ~36°41'36"N, 122°01'51"W, 439–814 m; coll. R/V ‘Velero IV’, stations 7462-61 or 7463-61, 10 October 1961; SBMNH 423075 [wet].

**Other material examined.** – 1 colony; USA, California, off San Francisco, Mulberry Seamount, ~37°26'48"N, 123°23'22"W, 1,255–1,455 m.; coll. N/B ‘Scofield’, location 38, 13 February 1950; CAS-IZ 96671 [wet]. --1 colony; USA, Alaska, Aleutian Islands, Adak Canyon, ~51°23'29"N, 177°07'38"W, 1,728 m; coll. R Stone using ROV ‘Jason II’, project ID # J2098-4-4, 29 July 2004; [dry].

***Muricellacomplanata* Wright & Studer, 1889**

**Material examined.** No confirmed identification of material in SBMNH collection.

**Other material examined** (Identification not confirmed). – 1 colony; USA, California, Santa Barbara County, Santa Barbara Channel, ~12 south of Santa Barbara, midway between Santa Barbara city and Santa Cruz island, oil platform (settling plates), ~34°13'39"N, 119°41'12"W, ~182 m; coll. MMS III Voucher, Survey, settling plates (incidental), station: exploratory wellsite Lease Number 0522, 6 February 1994; #0001942 [wet]. --2 colonies (2 lots); USA, California, Monterey County, Monterey Bay, Point Piños Light House, bearing S 3 degrees: distant 9.3 miles, ~36°46'14"N, 121°55'23"W, 518–649 m; coll. R/V ‘Albatross’, California Coastal Cruise 1904, Station #4461, 12 May 1904; CAS-IZ #s 5310 and 96669 [wet].
